# Cost-effectiveness of first-line encorafenib plus cetuximab with mFOLFOX6 in BRAF V600E-mutant metastatic colorectal cancer

**DOI:** 10.1016/j.isci.2026.114977

**Published:** 2026-02-10

**Authors:** Yamin Shu, Fenghao Shi, Jinlin Xiong, Jienan Zheng, Yiling Ding, Wenting Zhang, Pingping Xu, Qilin Zhang

**Affiliations:** 1 Department of Pharmacy, Tongji Hospital, Tongji Medical College, Huazhong University of Science and Technology, Wuhan 430030, China; 2 International Research Centre for Medicinal Administration, Peking University, Beijing 100191, China; 3 Department of Colorectal Surgery, Zhongshan Hospital, Fudan University, Shanghai 200032, China; 4 Department of Pharmacy, Union Hospital, Tongji Medical College, Huazhong University of Science and Technology, Wuhan 430022, China

**Keywords:** medical economics, pharmaceutical science, medical informatics, cancer, decision science

## Abstract

Patients with BRAF V600E-mutant metastatic colorectal cancer have poor prognosis, and the value of first-line targeted combination therapies remains uncertain. We evaluated the cost-effectiveness of encorafenib plus cetuximab with or without chemotherapy compared with standard chemotherapy, using clinical outcomes from a phase 3 randomized trial. A partitioned survival model with long-term extrapolation was applied to estimate lifetime costs and health outcomes from a United States payer perspective. Although both targeted regimens improved survival compared with standard chemotherapy, they were associated with substantially higher costs. Consequently, neither targeted strategy was cost-effective at commonly used willingness-to-pay thresholds under current pricing. These findings demonstrate that clinically meaningful survival gains may not translate into economic value in first-line settings. This study underscores the importance of incorporating economic evidence into early treatment adoption decisions and highlights the need for pricing approaches that better align costs with patient benefit.

## Highlights

• First-line EC ± mFOLFOX6 improved survival but was not cost-effective relative to SOC
• NNTs showed EC + mFOLFOX6 prevented progression and death, with early benefit
• Probabilistic analysis favored SOC as the most cost-effective strategy
• Value attribution revealed synergy but economic imbalance between components

## Graphical abstract

**Figure:**
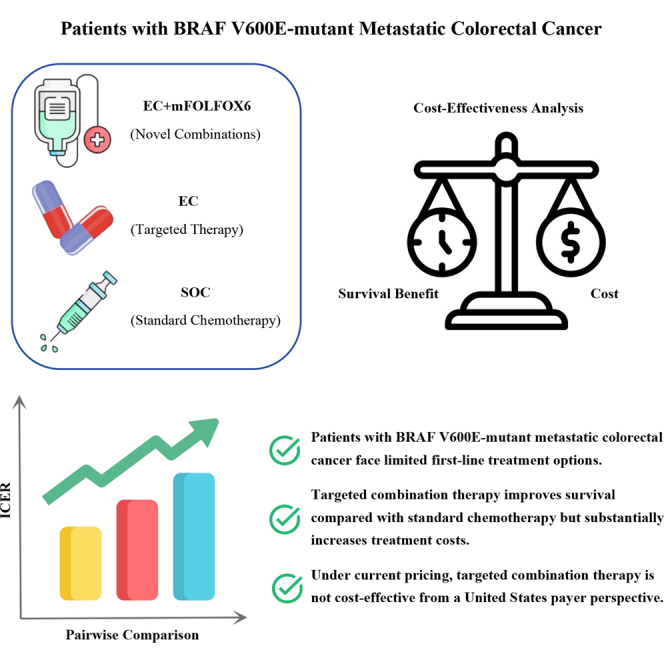

## Introduction

Colorectal cancer (CRC) is the second leading cause of cancer-related mortality and the third most common cancer globally.^1^ In 2022, 1.92 million CRC cases were diagnosed and 9,03,859 deaths due to CRC were reported worldwide, including 1,60,186 new cases and 54,614 deaths in the United States.^1^^,^^2^ BRAF V600E-mutated CRC represents a distinct subtype, which accounts for approximately 8%–12% of patients with metastatic disease.^3^^,^^4^ Despite intensive first-line regimens such as FOLFOXIRI plus bevacizumab, median overall survival (OS) remains limited to 15–17 months in the BRAF V600E-mutated CRC population, underscoring their poor prognosis compared with BRAF wild-type disease patients.^5^

Encorafenib is a highly specific competitive BRAF inhibitor with anti-proliferative and apoptotic activities in tumor cells carrying BRAF V600E mutations, exhibiting a more prolonged pharmacodynamic activity compared with other BRAF inhibitors.^4^^,^^6^ In CRC, suppressing the BRAF protein may trigger a swift feedback reactivation via epidermal growth factor receptors (EGFRs), thereby weakening its activity.^7^ Studies have shown that the combination of BRAF inhibitors with monoclonal antibodies targeting EGFRs enhances treatment efficacy compared with BRAF inhibition alone.^8^ Based on results from BEACON, encorafenib plus cetuximab was established as the second- or later-line treatment for BRAF V600E-mutant metastatic CRC (mCRC).^9^ However, in the first-line setting, the phase 2 ANCHOR study suggested only modest activity of encorafenib, binimetinib, and cetuximab relative to historical benchmarks, while the randomized FIRE-4.5 trial confirmed superior outcomes with FOLFOXIRI plus bevacizumab, underscoring the continuing importance of chemotherapy in this subtype.^5^^,^^10^

Recently, the phase 3 BREAKWATER trial evaluated first-line encorafenib plus cetuximab with or without chemotherapy (oxaliplatin, leucovorin, and 5-FU) (EC ± mFOLFOX6) vs. standard of care (SOC; chemotherapy with or without bevacizumab) in patients with BRAF V600E-mutant mCRC. The regimen EC + mFOLFOX6 demonstrated significant improvements in median progression-free survival (PFS, 12.8 vs. 7.1 months; hazard ratio [HR]: 0.53; 95% CI: 0.41–0.68; *p* < 0.0001) and OS (30.3 vs. 15.1 months; HR: 0.49; 95% CI: 0.38–0.63; *p* < 0.0001) compared with SOC.^4^ However, EC did not demonstrate a clear and robust incremental survival advantage over SOC in the first-line setting. Based on these results, EC + mFOLFOX6 was granted accelerated approval by the US Food and Drug Administration (FDA) as the first targeted therapy approved for first-line treatment of BRAF V600E-mutant mCRC.^11^

Although EC + mFOLFOX6 has demonstrated substantial improvements in survival outcomes, the introduction of a high-cost targeted combination at a first-line setting raises important concerns regarding value for money. Unlike later-line use, first-line adoption shifts a large proportion of lifetime treatment costs to the beginning of disease management, amplifying the budgetary and opportunity-cost implications for payers. Moreover, EC-based regimens consist of multiple active components with distinct costs and mechanisms of action, complicating the assessment of how incremental clinical benefit should be valued within a combination therapy framework. As first-line targeted strategies begin to reshape the treatment paradigm for BRAF V600E-mutant mCRC, robust economic evaluation is urgently needed to inform reimbursement decisions, pricing negotiations, and value-based policy discussions. Therefore, this study aimed to evaluate the cost-effectiveness of EC ± mFOLFOX6 compared with SOC as first-line therapy for BRAF V600E-mutant mCRC from a US payer perspective.

Although EC + mFOLFOX6 has demonstrated substantial improvements in survival outcomes, the introduction of a high-cost targeted combination in a first-line setting underscores the need for a formal assessment of its value for money. Unlike later-line use, first-line adoption shifts a substantial proportion of lifetime treatment costs to the early phase of disease management, with important implications for healthcare budgets and opportunity costs. Moreover, EC-based regimens comprise multiple active components with distinct costs and mechanisms of action, and their comparative economic value has not been directly assessed within a unified framework. As first-line targeted strategies begin to reshape the treatment paradigm for BRAF V600E-mutant mCRC, robust economic evaluation is needed to inform reimbursement decisions, pricing considerations, and value-based policy discussions. Accordingly, this study aimed to evaluate the cost-effectiveness of EC ± mFOLFOX6 compared with SOC as first-line therapy for BRAF V600E–mutant mCRC from a US payer perspective.

## Results

### Base-case analysis

Key model input parameters and base-case assumptions used in the analysis are summarized in Table 1. In the base-case analysis (Table 2), the EC + mFOLFOX6 regimen was associated with the highest total cost ($922,029), compared with EC ($560,796) and SOC ($229,219). Compared with EC, the addition of mFOLFOX6 increased costs by $361,234, while yielding 1.032 additional life-years (LYs) and 0.817 additional quality-adjusted life-years (QALYs). This resulted in an incremental cost-effectiveness ratio (ICER) of $442,033 per QALY. Relative to SOC, EC + mFOLFOX6 generated 1.797 more LYs and 1.376 more QALYs at an additional cost of $692,810, corresponding to an ICER of $503,391 per QALY. Similarly, EC versus SOC provided 0.765 more LYs and 0.559 more QALYs but at an incremental cost of $331576, resulting in an ICER of $593,077 per QALY. All ICERs were substantially above conventional willingness-to-pay (WTP) thresholds, leading to negative incremental net health benefit (INHB) and incremental net monetary benefit (INMB) values for both EC + mFOLFOX6 and EC compared with SOC. These findings suggest that neither EC + mFOLFOX6 nor EC alone would be considered cost-effective, and the addition of mFOLFOX6 to EC further increased costs, and, thus, could not achieve cost-effectiveness.

**Table 1:** Details of input parameters

Parameters	Mean	Lower limit	Upper limit	Distribution	α	β	Source
**Efficacy inputs of OS and PFS parametric survival curves**							–

EC + mFOLFOX6, OS, llogis, scale	3.32042	3.15199	3.48885	multivariate normal^a^	0.00738	−0.00351	–
EC + mFOLFOX6, OS, llogis, shape	0.52871	0.35246	0.70496		−0.00351	0.00809	–
EC + mFOLFOX6, PFS, llogis, scale	2.61476	2.46894	2.76059	multivariate normal^a^	0.00554	0.00554	–
EC + mFOLFOX6, PFS, llogis, shape	0.56027	0.41393	0.70662		−0.00132	0.00557	–
EC, OS, llogis, scale	3.01911	2.87412	3.16409	multivariate normal^a^	0.00547	−0.00125	–
EC, OS, llogis, shape	0.70457	0.53129	0.87784		−0.00125	0.00782	–
EC, PFS, llogis, scale	1.97733	1.82703	2.12764	multivariate normal^a^	0.00677	−0.00111	–
EC, PFS, llogis, shape	0.68934	0.52808	0.85060		−0.00111	0.00588	–
SOC, OS, gamma, rate	−2.36584	−2.63295	−2.09874	multivariate normal^a^	0.01857	0.01272	–
SOC, OS, gamma, shape	0.60967	0.40807	0.81127		0.01272	0.01058	–
SOC, PFS, lnorm, meanlog	2.02492	1.88357	2.16628	multivariate normal^a^	0.0052	0.00126	–
SOC, PFS, lnorm, sdlog	−0.05004	−0.17003	0.06996		0.00126	0.00375	–

**General settings**							

Average body surface area, m^2^	1.97	1.77	2.17	normal	–	–	Fryar. et al.^12^
Average weight, kg	83.35	75.02	91.68	normal	–	–	Kopetz. et al.^3^
Time horizon, years	30	5	30	uniform	–	–	National Institute for Health and Care Excellence (NICE)^13^
Discount rate	3%	0	0.06	uniform	–	–	Haacker. et al.^14^
Relative dose intensity of encorafenib	91.7%	80.49%	98.33%	beta	30.97	2.80	Kopetz. et al.^3^
Relative dose intensity of cetuximab	94.2%	81.79%	99.67%	beta	21.34	1.31	Kopetz. et al.^3^

**Costs**^b^**, $**							

Encorafenib per 75 mg	91.03	82.15	100.35	gamma	384.16	0.24	Micromedex.^15^
Cetuximab per 2 mg	16.73	15.10	18.44	gamma	384.16	0.04	Micromedex.^15^
Bevacizumab per 25 mg	199.24	179.81	219.64	gamma	384.16	0.52	Micromedex.^15^
Oxaliplatin per 5 mg	2.50	2.26	2.76	gamma	384.16	0.01	Micromedex.^15^
Leucovorin per 100 mg	24.09	21.74	26.56	gamma	384.16	0.06	Micromedex.^15^
5-FU per 1000 mg	10.28	9.28	11.33	gamma	384.16	0.03	Micromedex.^15^
Irinotecan per 100 mg	30.31	27.35	33.41	gamma	384.16	0.08	Micromedex.^15^
Capecitabine per 500 mg	1.02	0.92	1.12	gamma	384.16	0.00	Micromedex.^15^
I.V. push, add drug	54.26	48.97	59.82	gamma	384.16	0.14	Centers for Medicare & Medicaid Services^16^
I.V. infusion, up to 1 h	129.16	116.57	142.39	gamma	384.16	0.34	Centers for Medicare & Medicaid Services^16^
I.V. infusion, each additional hour	27.63	24.94	30.46	gamma	384.16	0.07	Centers for Medicare & Medicaid Services^16^
Prolonged chemo infusion >8 h	127.16	114.76	140.19	gamma	384.16	0.33	Centers for Medicare & Medicaid Services^16^
I.V. infusion, additional sequential infusion	63.58	57.38	70.09	gamma	384.16	0.17	Centers for Medicare & Medicaid Services^16^
BRAF V600E mutation testing	180.60	162.99	199.10	gamma	384.16	0.47	Centers for Medicare & Medicaid Services^17^
Tumor radiographic assessment	511.97	462.05	564.42	gamma	384.16	1.33	Centers for Medicare & Medicaid Services^16^
Consultant outpatient appointment	95.87	86.52	105.69	gamma	384.16	0.25	Centers for Medicare & Medicaid Services^16^
Subsequent treatment cost per week	2885.58	2604.21	3181.18	gamma	384.16	7.51	Kang. et al.^18^
End-of-life unit costs	31847.81	28742.32	35110.31	gamma	384.16	82.90	Aguiar-Ibáñez. et al.^19^

**Incidence of AEs in EC + mFOLFOX6**							**Elez. et al.**^4^

Anemia	15.1%	13.62%	16.64%	beta	326.00	1832.95	–
Asthenia	5.2%	4.69%	5.73%	beta	364.13	6638.40	–
Lipase increase	17.2%	15.51%	18.95%	beta	317.91	1530.42	–
Neuropathy peripheral	7.8%	7.04%	8.60%	beta	354.12	4185.85	–
Neutropenia	15.1%	13.62%	16.64%	beta	326.00	1832.95	–
Neutrophil count decrease	19.0%	17.14%	20.94%	beta	310.98	1325.76	–
Peripheral sensory neuropathy	6.9%	6.23%	7.61%	beta	357.58	4824.79	–

**Incidence of AEs in EC**							**Elez. et al.**^4^

Anemia	6.5%	5.87%	7.16%	beta	359.12	5165.87	–
Asthenia	0.7%	0.63%	0.77%	beta	381.46	54113.38	–
Lipase increase	3.3%	2.98%	3.64%	beta	371.45	10884.60	–
Neutropenia	1.3%	1.17%	1.43%	beta	379.15	28786.46	–
Neutrophil count decrease	0.7%	0.63%	0.77%	beta	381.46	54113.38	–

**Incidence of AEs in SOC**							**Elez. et al.**^4^

Anemia	3.9%	3.52%	4.30%	beta	369.14	9095.96	–
Asthenia	1.3%	1.17%	1.43%	beta	379.15	28786.46	–
Lipase increase	6.1%	5.50%	6.72%	beta	360.67	5551.88	–
Neuropathy peripheral	3.5%	3.16%	3.86%	beta	370.68	10220.16	–
Neutropenia	10.0%	9.02%	11.02%	beta	345.64	3110.80	–
Neutrophil count decrease	17.0%	15.33%	18.73%	beta	318.68	1555.92	–
Peripheral sensory neuropathy	3.5%	3.16%	3.86%	beta	370.68	10220.16	–

**AE cost**^b^**, $**							**Centers for Medicare & Medicaid Services**^20^

Anemia	6,816	6,151	7,514	gamma	384.16	17.74	–
Asthenia	6,816	6,151	7,514	gamma	384.16	17.74	–
Lipase increase	7,609	6,867	8,389	gamma	384.16	19.81	–
Neuropathy peripheral	7,773	7,015	8,569	gamma	384.16	20.23	–
Neutropenia	12,243	11,049	13,497	gamma	384.16	31.87	–
Neutrophil count decrease	12,243	11,049	13,497	gamma	384.16	31.87	–
Peripheral sensory neuropathy	7,773	7,015	8,569	gamma	384.16	20.23	–

**Disutilities for AEs**							

Anemia	0.09	0.081	0.099	beta	349.50	3,533.79	NICE^21^
Asthenia	0.115	0.104	0.127	beta	339.87	2,615.50	NICE^21^
Lipase increase	0.08	0.072	0.088	beta	353.35	4,063.49	NICE^22^
Neuropathy peripheral	0.075	0.068	0.083	beta	355.27	4,381.70	NICE^23^
Neutropenia	0.0607	0.055	0.067	beta	360.78	5,582.89	NICE^22^
Neutrophil count decrease	0.0375	0.034	0.041	beta	369.72	9,489.39	NICE^22^
Peripheral sensory neuropathy	0.075	0.068	0.083	beta	355.27	4,381.70	NICE^23^

**Health utility**							

PFS	0.8	0.776	0.822	beta	888.09	222.02	NICE^24^
PD	0.73	0.698	0.761	beta	561.31	207.61	NICE^24^

**Disutilities prior to death**							**Borseet al.**^25^

90–180 days	0.03438	0.031	0.038	beta	370.92	10,417.86	–
30–90 days	0.1105	0.100	0.122	beta	341.60	2,749.80	–
0–30 days	0.2259	0.204	0.249	beta	297.15	1,018.26	–

Abbreviations: OS, overall survival; PFS, progression-free survival; PD, progressed disease; EC + mFOLFOX6, encorafenib and cetuximab plus oxaliplatin, leucovorin, and 5-FU; SOC, standard of care; 5-FU, 5-fluorouracil; I.V., intravenous; AEs, adverse events; NICE, National Institute for Health and Care Excellence.

a For parameters modeled under a multivariate normal distribution, the results are expressed as variance-covariance matrices, in place of separate α and β estimates.

b Economic inputs were converted to July 2025 US dollars by using consumer price index (CPI)-based inflation adjustments.

**Table 2:** Base-case results for costs and health outcomes

Variables	EC + mFOLFOX6	EC	SOC	Incremental		
				EC + mFOLFOX6 vs. EC	EC + mFOLFOX6 vs. SOC	EC vs. SOC
Total costs, $	922,029	560,796	229,219	361,234	692,810	331,576
Drug acquisition	597,605	293,373	74,338	304,232	523,267	219,035
Drug administration	26,727	3114	10,172	23,612	16,555	−7,058
Subsequent treatment	241,889	220,169	95,427	21,720	146,461	124,741
Monitoring	12,117	6459	6773	5,658	5,343	−314
Adverse events	8,429	1038	4912	7,390	3,516	−3,874
End of life	35,264	36,642	37,596	−1,378	−2,332	−954
Total LYs, year	3.368	2.336	1.572	1.032	1.797	0.765
PFS	1.796	0.905	0.951	0.891	0.844	−0.046
PD	1.573	1.432	0.620	0.141	0.952	0.811
Total QALYs, QALY	2.584	1.767	1.208	0.817	1.376	0.559
PFS	1.437	0.723	0.760	0.714	0.677	−0.037
PD	1.151	1.047	0.454	0.104	0.697	0.594
Adverse event disutilities	0.00116	0.00020	0.00053	0.00096	0.00063	−0.00033
Time-to-death disutilities	0.00254	0.00291	0.00514	−0.00036	−0.00259	−0.00223
Incremental costs per LY, $	–	–	–	350,072	385,586	433,496
Incremental costs per QALY, $	–	–	–	442,033	503,391	593,077
INHB, QALY	–	–	–	−1.591	−3.242	−1.651
INMB, $	–	–	–	−238,652	−486,367	−247,715
EVPI/person, $	–	–	–	0		

Abbreviations: PFS, progression-free survival; PD, progressed disease; EC + mFOLFOX6, encorafenib and cetuximab plus oxaliplatin, leucovorin and 5-FU; SOC, standard of care; INHB, incremental net health benefit; INMB, incremental net monetary benefit; QALYs, quality-adjusted life-years; EVPI, expected value of perfect information.

### Sensitivity analyses

**Figure 1:**
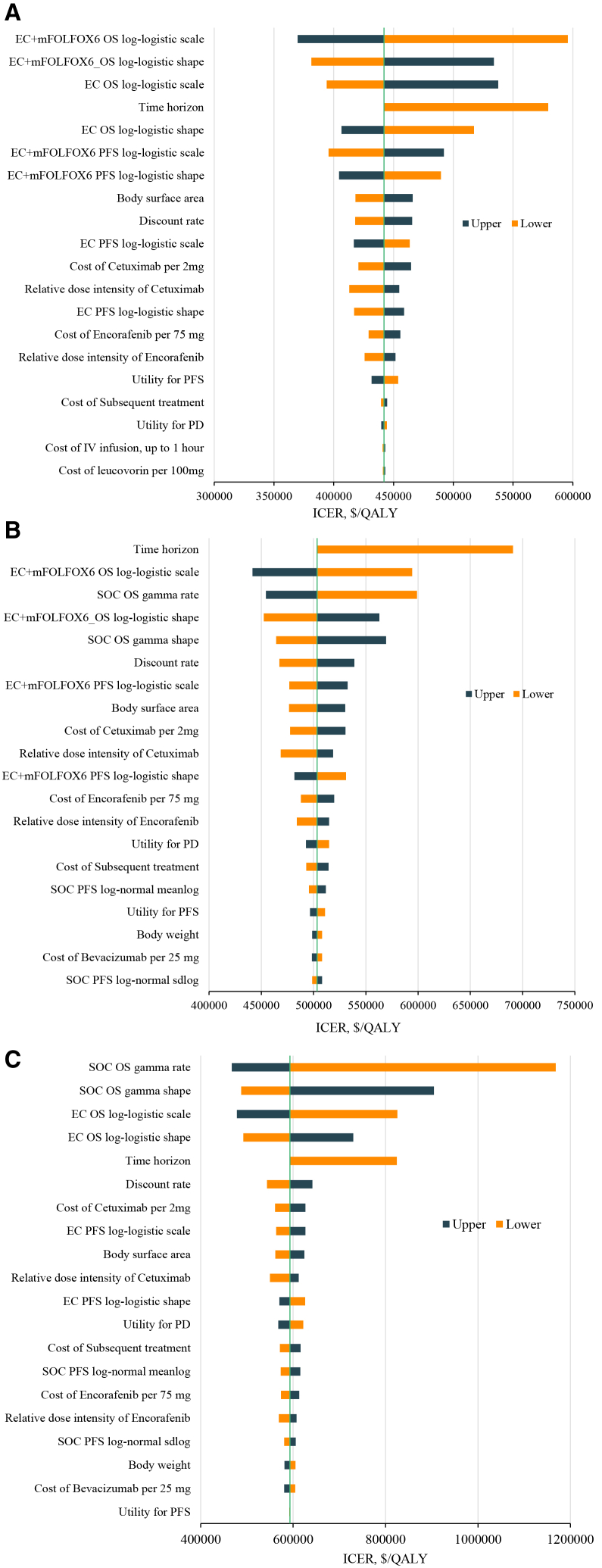
Tornado diagrams of one-way sensitivity analyses (A) EC + mFOLFOX6 vs. EC. (B) EC + mFOLFOX6 vs. SOC. (C) EC vs. SOC. Abbreviations: PFS, progression-free survival; PD, progressed disease; EC + mFOLFOX6, encorafenib and cetuximab plus oxaliplatin, leucovorin, and 5-FU; SOC, standard of care; QALY, quality-adjusted life-years; ICER, incremental cost-effectiveness ratio.

One-way sensitivity analyses for EC + mFOLFOX6 vs. EC, EC + mFOLFOX6 vs. SOC, and EC vs. SOC (Figure 1) showed that parameters related to survival extrapolation were the primary sources of uncertainty. Specifically, the log-logistic shape and scale parameters for PFS and OS in the EC + mFOLFOX6 and EC arms, together with the gamma shape and rate for OS in SOC, produced the largest variation in ICERs. Following the extrapolation parameters, time horizon and discount rate emerged as major determinants of cost-effectiveness outcomes. Extending the analytic horizon or lowering the discount rate consistently improved QALY gains, thereby reducing ICERs, while shorter horizons or higher discount rates diminished long-term benefits. Other influential factors included drug-related parameters, notably the relative dose intensities and costs of encorafenib and cetuximab. Patient-level characteristics such as body surface area and weight, as well as health-state utilities for PFS and progressed disease (PD), also contributed to ICER variability. In contrast, costs of leucovorin, infusion, and subsequent treatments had only marginal effects.

The two-way sensitivity analysis further illustrated the joint impact of drug prices on cost-effectiveness. As shown in Figure S1, EC + mFOLFOX6 became the preferred strategy over SOC only when the costs of both encorafenib and cetuximab were simultaneously reduced to extremely low levels, whereas EC could not achieve cost-effectiveness under any tested price scenarios. Across the majority of the parameter space, SOC remained the optimal strategy, highlighting that substantial price reductions in both targeted agents would be required for EC + mFOLFOX6 to achieve cost-effectiveness.

**Figure 2:**
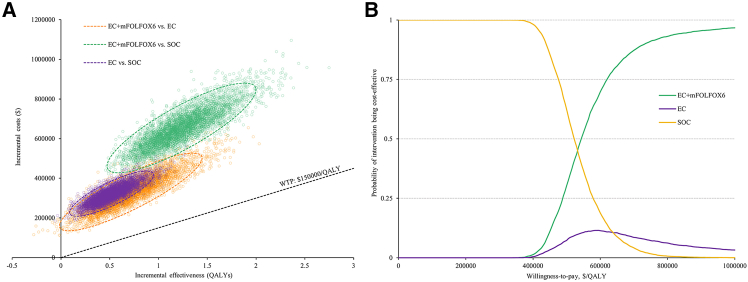
Results of the probabilistic sensitivity analysis (A) Scatterplot on the cost-effectiveness plane. (B) Cost-effectiveness acceptability curve. Abbreviations: EC + mFOLFOX6, encorafenib and cetuximab plus oxaliplatin, leucovorin, and 5-FU; SOC, standard of care; QALY, quality-adjusted life-years; WTP, willingness-to-pay.

The probabilistic sensitivity analysis (PSA) confirmed the robustness of the base-case findings (Table S1). Compared with EC, EC + mFOLFOX6 incurred substantially higher costs (mean $883,919; 95% CI: 718,094–1,087,598 vs. $552,403; 95% CI: 470,878–653,996) with modest QALY gains (2.45 vs. 1.73; incremental 0.68 QALYs; 95% CI: 0.40–1.37), yielding a mean ICER of $507,586 per QALY (95% CI: 326,859–1,046,336). Against SOC, EC + mFOLFOX6 produced 1.61 additional QALYs (95% CI: 0.92–2.93) but at an incremental cost of $654,375 (95% CI: 492,580–854,772), resulting in an ICER of $548,137 per QALY (95% CI: 412,764–758,836). Similarly, EC vs. SOC was associated with a mean ICER of $680,325 per QALY (95% CI: 433,221–1,218,093). At a WTP of $150,000 per QALY, SOC had nearly 100% probability of being the most cost-effective strategy, while EC + mFOLFOX6 and EC showed negligible probabilities (Figure 2A). Only when the WTP exceeded approximately $400,000/QALY, did EC + mFOLFOX6 begin to gain probability, surpassing SOC beyond $500,000/QALY (Figure 2B). In contrast, EC never emerged as the preferred strategy at any WTP level. The expected value of perfect information (EVPI) per patient was estimated to be zero at the WTP threshold, indicating that eliminating parameter uncertainty would not provide additional value for decision-making. These findings indicate that SOC is overwhelmingly the most cost-effective option, and substantial increases in WTP would be required before EC + mFOLFOX6 could be considered economically favorable.

Scenario analyses yielded results consistent with those of the base-case analyses (Table S2). Under alternative survival modeling approaches, including spline, Akaike information criterion (AIC)-weighted, and Bayesian information criterion (BIC)-weighted models (scenarios 1–3), EC + mFOLFOX6 versus SOC produced incremental QALY gains ranging from 0.93 to 1.17, with corresponding ICERs between $463,074 and $578,029 per QALY. Compared with EC, the incremental QALY gains for EC + mFOLFOX6 ranged from 0.56 to 0.72, with ICERs spanning $440,725 to $451,450 per QALY. When utilities were restricted to progression-based health states (scenario 4), ICERs remained essentially unchanged relative to the base case (EC + mFOLFOX6 vs. SOC: $504,038/QALY). Excluding relative dose intensity assumptions (scenario 5) slightly increased costs, leading to higher ICERs (EC + mFOLFOX6 vs. SOC; $534,036/QALY).

### Numbers needed to treat and value attribution

The results for the numbers needed to treat (NNTs) to prevent one progression or death event at different time points across treatment comparisons are summarized in Tables S3 and S4. At 6 months, the NNT to prevent one additional progression event was 4.9 (95% CI: 3.4–8.4) for EC + mFOLFOX6 vs. SOC and 3.4 (95% CI: 2.5–5.2) for EC + mFOLFOX6 vs. EC, while EC vs. SOC showed no benefit (NNT: –11.1; 95% CI: –48.4 to −5). The NNT to prevent one additional death event at 6 months was 16.9 (95% CI: 9.0–150.0) for EC + mFOLFOX6 vs. SOC; 500.0 (95% CI: 19.0 to −20.6) for EC + mFOLFOX6 vs. EC; and 17.5 (95% CI: 8.7–1,775.4) for EC vs. SOC. At 1 year, the NNTs for preventing progression events remained favorable for EC + mFOLFOX6 (4.0; 95% CI: 2.9–6.8 vs. SOC; 3.8; 95% CI: 2.7–6.4 vs. EC), whereas EC vs. SOC continued to be unfavorable (−58.8; 95% CI: –10.7 to −7.8). For preventing death events, the NNTs were 7.0 (95% CI: 4.5–15.9) for EC + mFOLFOX6 vs. SOC, 14.5 (95% CI: 6.4–52.9) for EC + mFOLFOX6 vs. EC, and 13.5 (95% CI: 5.9–49.7) for EC vs. SOC. At 2 years, EC + mFOLFOX6 consistently prevented additional progression events with NNTs of 5.3 (95% CI: 3.3–12.9) vs. SOC and 4.5 (95% CI: 3.1–8.7) vs. EC, while those for EC vs. SOC remained negative (−33.3; 95% CI: –12.5 to −7.2). For death events, the NNTs were 4.1 (95% CI: 2.9–7.4) for EC + mFOLFOX6 vs. SOC, 7.9 (95% CI: 4.2–69.5) for EC + mFOLFOX6 vs. EC, and 8.8 (95% CI: 4.5–206.5) for EC vs. SOC. These findings indicate that the addition of mFOLFOX6 to EC substantially reduced the risk of both progression and death events, achieving low and clinically meaningful NNT values as early as 6 months and maintaining benefit until 1 and 2 years, whereas EC alone did not provide consistent benefit compared with SOC.

**Figure 3:**
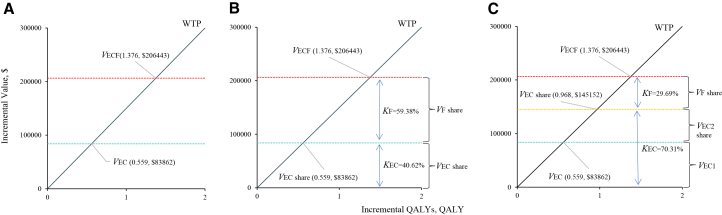
Results of the value attribution analysis across alternative scenarios (A) Unallocated. (B) Imperfect information with balanced market power. (C) Imperfect information with imbalanced market power. Abbreviations: WTP, willingness-to-pay; QALY, quality-adjusted life-years; ECF, encorafenib and cetuximab plus oxaliplatin, leucovorin, and 5-FU; EC, encorafenib and cetuximab; *V*, monetary value; *K*, proportion of the value attributed to the combination treatment.

Figure 3 illustrates the value attribution analysis under different assumptions at a WTP threshold of $150,000 per QALY. Figure 3A shows that the total incremental value of EC + mFOLFOX6 compared with SOC was 1.376 QALYs and $206,443, of which EC alone contributed 0.559 QALYs, equivalent to $83,862. When applying the imperfect information framework with balance of market power (Figure 3B), the incremental value was allocated between mFOLFOX6 and EC. Under this assumption, 59.4% of the total value was attributed to mFOLFOX6 (0.817 QALYs; $122,581), while 40.6% was attributed to EC (0.559 QALYs; $83,862). In contrast, under imperfect information with imbalance of market power (Figure 3C), the share of value shifted substantially. EC was assigned 70.3% of the total incremental value (0.968 QALYs; $145,152), whereas mFOLFOX6 accounted for only 29.7% (0.408 QALYs; $61,291).

## Discussion

This economic evaluation provides a comprehensive assessment of first-line strategies for patients with BRAF V600E-mutant mCRC, and to our knowledge, represents the first study to evaluate the cost-effectiveness of both doublet (EC) and triplet (EC + mFOLFOX6) targeted regimens in the US. Despite demonstrating meaningful clinical benefits, particularly in terms of reduced NNTs for progression and death, both EC and EC + mFOLFOX6 failed to achieve cost-effectiveness compared with SOC, with ICERs substantially exceeding the conventional WTP threshold of $150,000 per QALY. These findings highlight the persistent challenge in oncology of reconciling innovation-driven survival gains with affordability.

Clinical evidence helps explain these outcomes. The results from the ANCHOR trial demonstrated that the targeted triplet regimen of encorafenib, cetuximab, and binimetinib was active in the first-line setting but conferred only a limited PFS benefit, inferior to that achieved with FOLFOXIRI plus bevacizumab, suggesting that EC alone is unlikely to demonstrate superiority over standard chemotherapy.^10^ In this context, the BREAKWATER design sought to integrate chemotherapy’s rapid cytoreductive effect with the more durable disease control of EC, creating a complementary strategy.^3^ Our NNT analysis demonstrated that EC vs. SOC did not provide consistent clinical advantage, with negative or unstable NNT values across multiple time points. By contrast, the addition of mFOLFOX6 to EC markedly reduced the NNT, reaching approximately 4–5 for progression events at 6–12 months and 7.0 for death events at 12 months, indicating a clinically meaningful benefit. These findings reinforce that while EC alone is unlikely to demonstrate superiority over SOC, the triplet regimen provides complementary clinical benefit through the combined effects of targeted therapy and chemotherapy. Indeed, EC + mFOLFOX6 generated an incremental 1.376 QALYs compared with SOC, whereas EC alone achieved only 0.559 QALYs. Although the direct incremental benefit of mFOLFOX6 compared with SOC could not be calculated, it is unrealistic to assume that upgrading SOC chemotherapy to mFOLFOX6 alone would yield a net increase of 0.817 QALYs. This strongly suggests a true synergistic effect between chemotherapy and targeted therapy, with chemotherapy driving early response and EC maintaining long-term disease control. Despite this clinical synergy, economic results remained unfavorable. The triplet regimen incurred markedly higher costs, and ICERs for all pairwise comparisons were well above the accepted thresholds. Sensitivity analyses identified survival extrapolation parameters, time horizon, and drug acquisition costs as the main drivers of cost-effectiveness outcomes, while probabilistic simulations confirmed an almost zero probability of cost-effectiveness at conventional WTP thresholds. The EVPI was estimated to be zero, which indicates that further reduction in parameter uncertainty would not change decision-making under current pricing structures.

The value attribution analysis provides further context for this paradox. Prior cost-effectiveness evaluations of EC in the second-line BEACON trial revealed the so-called “not cost-effective at zero price” phenomenon.^26^ Even when encorafenib was hypothetically priced at zero, EC remained less economical than chemotherapy, largely because cetuximab itself is not cost-effective in mCRC.^26^ Extending cetuximab use into earlier treatment lines amplifies this problem, as the incremental survival gains from EC are insufficient to compensate for the cost burden of cetuximab. In our study, EC was defined as the backbone regimen and mFOLFOX6 as the add-on. Under balanced market power, incremental QALY value was primarily attributed to the add-on component, while under imbalanced assumptions, a greater share of value accrued to the backbone component, with EC capturing the larger proportion.^27^ This highlights the difficulty of allocating value between the targeted and chemotherapy components when one drug is clinically relevant but economically unfavorable. Taken together, these findings indicate that, under current US pricing, SOC remains the most economically efficient first-line strategy for BRAF V600E-mutant mCRC. While neither EC nor EC + mFOLFOX6 can currently be justified on cost-effectiveness grounds, this evaluation highlights broader methodological and policy considerations. In particular, the application of advanced survival modeling, the integration of clinically meaningful measures such as NNT, and the exploration of structured value attribution frameworks provide valuable insights for assessing high-cost oncology regimens and informing price negotiations. Although this evaluation was conducted from a US payer perspective, the findings offer broader insights for health systems facing similar decisions regarding the adoption of high-cost targeted combinations in first-line settings. In jurisdictions with lower WTP thresholds or centralized price negotiation mechanisms, the magnitude of incremental costs identified in this study underscores the potential need for price reductions, indication-specific pricing, or value-based agreements to improve affordability. Importantly, the analytic framework applied here may support international reimbursement and pricing discussions by clarifying the relationship between clinical benefit, cost burden, and value attribution in multicomponent regimens.

### Limitations of the study

Several limitations should be acknowledged, which also indicate important directions for future research. First, although survival curves were reconstructed using validated methods and aligned with published Kaplan-Meier data, long-term projections remain uncertain without mature follow-up or real-world evidence beyond five years. External validation using MD Anderson and Surveillance, Epidemiology, and End Results (SEER) cohorts increased plausibility but should be interpreted with caution, as survival outcomes may be influenced by differences in patient characteristics, treatment settings, and calendar periods. Future studies should integrate real-world datasets stratified by the mutation status and treatment history to improve external validity. Second, health state utilities and cost parameters were primarily derived from published sources and US payer databases, which may not fully capture heterogeneity in patient experiences or variations in clinical practice. Our model incorporated multiple determinants of utility and applied age-adjusted decrements over time to reflect the natural decline in health-related quality of life (HRQoL). Nevertheless, this approach may not fully capture treatment-specific differences in terms of QoL impact. In addition, adverse event (AE) disutilities and post-progression therapy costs were modeled based on published sources and simplifying assumptions. In practice, the incidence, duration, and severity of AEs, as well as the intensity and composition of post-progression treatments, may vary across patients and clinical settings. Although these parameters were explored through sensitivity analyses and not identified as primary drivers of cost-effectiveness outcomes, residual uncertainty may, nonetheless, influence the robustness of ICER estimates. Future clinical trials should systematically collect patient-reported outcomes to provide more granular utility estimates, while real-world studies documenting subsequent therapy use, costs, and QoL trajectories would further enhance the generalizability and robustness of future models. Third, the value attribution framework, although methodologically informative, was based on stylized assumptions regarding market power that may not accurately reflect the complexity of real-world, payer-manufacturer negotiations. In practice, price setting is influenced by regulatory policies, confidential rebates, and negotiation dynamics that extend beyond theoretical allocation rules. Methodological refinement is, therefore, required to develop value attribution approaches that can more realistically accommodate multicomponent regimens, particularly in scenarios where one drug is clinically effective yet economically unfavorable. In summary, addressing these limitations through longer-term evidence, prospective measurement of HRQoL, improved cost documentation, methodological innovation in value attribution, and more robust real-world validation will be essential to strengthen the evidence base and guide sustainable adoption of targeted regimens in BRAF V600E-mutant mCRC.

## Resource availability

### Lead contact

Requests for further information and resources should be directed to and will be fulfilled by the lead contact, Qilin Zhang (qilinzhang88@163.com).

### Materials availability

The study did not generate new materials.

### Data and code availability

• This article analyzes existing, publicly available data. All data sources are indicated in the manuscript and supplemental files.
• This paper does not report original code.
• This paper does not report any additional resources.

## Acknowledgments

The authors gratefully acknowledge the financial support from Project of the 10.13039/100017958Health Commission of Hubei Province (WJ2025M193), Talent Project established by 10.13039/501100004433Chinese Pharmaceutical Association Hospital Pharmacy Department (CPA-Z05-ZC-2023-003), and Dawning Program of Wuhan Knowledge Innovation Special Project (2023020201020501).

## Author contributions

Conceptualization, Y.S., F.S., P.X., and Q.Z.; methodology, Y.S., F.S., J.X., J.Z., and Q.Z.; investigation, Y.D., W.Z., and P.X.; writing – original draft, Y.S., F.S., P.X., and Q.Z.; writing – review & editing, all authors; funding acquisition, Q.Z.; resources, J.X., J.Z., and P.X.; supervision, P.X., W.Z., and Y.D.

## Declaration of interests

The authors declare no competing interests.

## STAR★Methods

### Key resources table

**Table:** 

REAGENT or RESOURCE	SOURCE	IDENTIFIER
**Deposited data**		

Clinical data	BREAKWATER trial	https://www.nejm.org/doi/full/10.1056/NEJMoa2501912
Drug cost data	RED BOOK	https://www.micromedexsolutions.com

**Software and algorithms**		

R (version 4.4.2)	R Core Team	https://www.r-project.org
RStudio (version 2024.12.0)	Posit Software	https://posit.co/products/open-source/rstudio/
Microsoft Excel (version 2511)	Microsoft Corporation	https://www.microsoft.com/microsoft-365/excel

### Experimental model and study participant details

This study did not involve human or animal subjects. Our research data are derived from the published BREAKWATER trial (NCT04607421) enrolled in 28 countries and other public databases and literature.

### Method details

#### Participants and therapeutic interventions

The target population comprised adults with BRAF V600E-mutant metastatic mCRC receiving first-line therapy, consistent with the intention-to-treat population of the BREAKWATER trial and the FDA-approved indication.^3^^,^^4^^,^^11^ The mean age of patients was 61 years, with an equal distribution of males and females. According to the National Center for Health Statistics (NCHS) data,^12^ an average weight of 83.35 kg and height of 1.68 m were used to derive a mean body surface area (BSA) of 1.97 m^2^ for dose calculation. Patients were randomized (1:1:1) to receive: EC: encorafenib 300 mg orally once daily + cetuximab 500 mg/m^2^ intravenously every 2 weeks; EC + mFOLFOX6: EC plus oxaliplatin 85 mg/m^2^ IV, leucovorin 400 mg/m^2^ IV, 5-FU 400 mg/m^2^ IV bolus, followed by 5-FU 2400 mg/m^2^ continuous IV infusion over 46–48 h, repeated every 2 weeks (28-day cycle); SOC: investigator’s choice of mFOLFOX6, FOLFOXIRI, or CAPOX, each with or without bevacizumab. All treatments were continued until disease progression, unacceptable toxicity, withdrawal, or death. This study was conducted in accordance with the Consolidated Health Economic Evaluation Reporting Standards (CHEERS) guideline for economic evaluations.^28^

#### Model structure

The economic evaluation employed an area under the curve (AUC) partitioned survival model (PSM) with three mutually exclusive health states: PFS, progressed disease (PD), and death (Figure S2). Health state occupancy over time was derived from trial survival curves: PFS(t) from the PFS curve, PD(t) as OS(t)-PFS(t), and Death(t) as 1-OS(t).^29^ Progression-based models are widely used in economic evaluations of oncology treatments,^30^ as they capture the progressive nature of disease through distinct pre- and post-progression states and mirror the clinical care pathway in mCRC. This structure reflects the endpoints of the BREAKWATER trial (PFS and OS) and captures the time-dependent risk of events by modeling survival as a function of time since model entry, avoiding additional structural assumptions required to estimate transition probabilities in a state-transition model.^31^ This approach has also been frequently applied in health technology assessment (HTA) submissions to the National Institute for Health and Care Excellence (NICE). At model initiation, all patients were assumed to be in the PFS state and received EC + mFOLFOX6, EC, or SOC. As the simulation advanced, patients could remain PFS, transition directly to death, or move into the PD state, where subsequent treatments were administered before eventual transition to death. The PFS and OS curves from the BREAKWATER trial were extracted by GetData Graph Digitizer and transformed into pseudo-individual patient data (IPD) using the reconstruction approach of Guyot et al.^32^ The accuracy of the reconstructed curves was evaluated through visual inspection of their concordance with the published Kaplan-Meier curves and by contrasting principal survival endpoints, with detailed results illustrated in Figures S3, S4, and Table S5.

Per NICE guidance,^13^ a lifetime horizon should be used to capture all cost and outcome differences between comparators; therefore, a 30-year horizon was applied in the base-case analysis for first-line mCRC. Weekly cycles, together with half-cycle correction, were applied in the model to reflect different treatment schedules and to achieve adequate precision. A range of survival extrapolation methods was applied, comprising seven standard distributions (exponential, Weibull, Gompertz, log-logistic, log-normal, gamma, and generalized gamma, Figure S5), flexible spline-based models with 1, 2, or 3 knots (Figure S6), and model averaging based on Akaike information criterion (AIC)- and Bayesian information criterion (BIC)-weighted estimates (Figures S7 and S8).^33^ For each endpoint, the preferred parametric distribution was determined through evaluation of statistical goodness-of-fit (Table S6), inspection of visual agreement with observed data, validation of long-term projections by clinical experts, and where possible, comparison against published real-world evidence. In a retrospective cohort study conducted at MD Anderson Cancer Center including 1420 patients with mCRC, the 3-year OS rate among those with BRAF V600E mutations improved from 19.6% in 2010–2015 to 37.6% in 2016–2019, with the median OS increasing from 13.9 months to 35.2 months^34^ Based on Surveillance, Epidemiology, and End Results (SEER) database, the 5-year relative survival rate for patients with mCRC is approximately 13–18% in 2014–2020, which reflects the overall mCRC population and is not specific to BRAF V600E mutations.^35^ Specifically, outcomes reported from MD Anderson Cancer Center and the SEER database were used as external plausibility checks for long-term OS projections, rather than as model inputs, to complement statistical goodness-of-fit and visual inspection when selecting preferred extrapolation models. Table S7 reported parameter estimates for all extrapolation models. The proportional hazards (PH) assumption was assessed using log[-log(S(t))] plots (Figures S9 and S10); however, the log-cumulative hazard curves showed clear crossing, indicating a violation of the PH assumption. Accordingly, separate parametric survival models were independently fitted to each treatment group for extrapolation of all relevant endpoints. The impact of parameter distributions on predicted hazards is demonstrated in Figure S11. Survival analyses were carried out in R, whereas the PSM was developed in Microsoft Excel to facilitate transparent implementation.

In the base-case analysis, the best-fitting conventional parametric distributions were selected through a comprehensive evaluation (Table 1): log-logistic models for PFS and OS in the EC and EC + mFOLFOX6 arms, a log-normal model for SOC PFS, and a gamma model for SOC OS. Both costs and health outcomes were discounted at an annual rate of 3%.^14^ Pairwise comparisons were performed among EC + mFOLFOX6, EC, and SOC. The model generated point estimates for life-years (LYs), quality-adjusted life-years (QALYs), total costs, incremental cost-effectiveness ratios (ICERs), incremental net health benefit (INHB), and incremental net monetary benefit (INMB). The willingness-to-pay (WTP) threshold was set at $150000 per QALY.^36^

#### Costs and utilities

Direct medical costs were estimated from the US payer perspective and encompassed drug acquisition, administration, disease monitoring, management of treatment-related AEs, subsequent therapies, and terminal care. Drug acquisition costs for each treatment were calculated by multiplying unit prices from the REDBOOK by the dosing schedules reported in the BREAKWATER trial.^15^ The relative dose intensity of encorafenib (91.7%) and cetuximab (94.2%), as reported in the trial,^3^ was incorporated into the base-case model to account for missed doses and dose reductions. Drug administration costs were calculated using unit costs from the 2024 Centers for Medicare & Medicaid Services (CMS) fee schedules,^16^ with infusion duration, method, and sequence aligned with the trial protocol. Disease monitoring costs included BRAF V600E mutation testing, sourced from CMS Molecular Pathology reimbursement schedules,^17^ as well as tumor radiographic assessments (e.g., CT scans) and consultant outpatient appointments, valued using CMS fee schedules.^16^ The cost of managing treatment-related AEs was estimated by multiplying the incidence of each AE by its unit cost per episode and summing across all included events. These costs were applied as a one-off expense at model initiation. Only grade 3 or 4 AEs with an incidence of at least 5% in either arm were incorporated. Unit costs for AE management were derived from diagnosis-related group (DRG).^20^ Expenditures related to subsequent lines of therapy were obtained from a published US-based cost-effectiveness study in mCRC.^18^ Terminal care costs were derived from an analysis of end-of-life health care utilization and expenditures among oncology patients covered by a US commercial insurance database.^19^ Before being entered into the model, all costs reported in Table 1 for years prior to 2025 were inflated to July 2025 US dollars using the Consumer Price Index (CPI) published by the US Bureau of Labor Statistics.

Clinical expert opinion suggested that health-related quality of life (HRQoL) in patients with mCRC is driven primarily by disease progression status rather than by the treatment received.^37^ Accordingly, identical utility values were applied across treatment arms within both the PFS and PD health states. Progression-based health state utility values for mCRC were sourced from NICE TA709,^24^ with utilities of 0.80 for PFS and 0.73 for PD. The base-case model also incorporated the impact of AEs on HRQoL. Health disutilities from AEs were calculated as the product of the expected utility decrement and the duration of each event. Estimates of AE-related disutilities were derived from published studies,^21^^,^^22^^,^^23^ with the severity of most events assumed to diminish substantially within 1 week. Following NICE guidance, utility decrements over the model horizon were applied to age- and sex-adjusted general population norms (Figure S12), with background utilities estimated using the algorithm proposed by Ara et al.^38^ At model initiation, the baseline general population utility was 0.822 (age 61 years, 50% male). Utility multipliers (0.973 for PFS and 0.888 for PD) were derived and applied to age-adjusted general population utilities, which were updated each cycle to produce time-varying utilities for the PFS and PD states across all treatments. Moreover, end-of-life disutilities were applied at new death, derived from a mixed-effects linear regression of patient-level EQ-5D-3L data and valued using the US societal tariff.^25^

#### Sensitivity analysis

Model robustness was examined through deterministic sensitivity analysis (DSA), probabilistic sensitivity analysis (PSA), and scenario analyses. In the DSA, variations in single parameters or selected parameter pairs across plausible ranges were explored to assess their effect on the ICER. Parameters were varied sequentially within their 95% confidence intervals (CIs); where CIs were unavailable, a ±10% range around the base-case value was assumed to capture plausible uncertainty. Standard errors were derived by back-calculating from the 95% CI under the assumption of a normal distribution. In the PSA, parameters were assigned appropriate probability distributions (multivariate normal for extrapolation, beta for probabilities and utilities, gamma for costs, *etc*.) and jointly varied. To account for correlations among extrapolation parameters, the variance-covariance matrix was decomposed using the Cholesky method,^39^^,^^40^ producing a lower triangular matrix that enabled correlated sampling within the PSA (Table S8). Model uncertainty was assessed using 5000 Monte Carlo simulations, and outcomes were summarized on the cost-effectiveness plane (CEP) and through cost-effectiveness acceptability curves (CEACs) across varying WTP thresholds. Based on the iterative simulation results, the expected value of perfect information (EVPI) was estimated for EC + mFOLFOX6, EC, and SOC to assess the value of eliminating decision uncertainty among these mutually exclusive first-line strategies. EVPI was derived as: Average (Max (NMB, EC + mFOLFOX6, EC, and SOC)) - Max (Average (NMB, EC + mFOLFOX6, EC, and SOC)). Additional scenario analyses were performed to test the sensitivity of the base-case results to key structural and methodological assumptions, including the use of spline model, AIC-weighted model, BIC-weighted model, progression-based utilities only, and exclusion of relative dose intensity.

#### Number needed to treat and value attribution

The number needed to treat (NNT) represents the number of patients who must receive a given therapy to prevent one additional progression or death and provides an absolute, clinically interpretable summary of treatment benefit.^41^^,^^42^ In this study, NNT was calculated as a supplementary measure to contextualize the cost-effectiveness results, rather than as a prespecified primary outcome. As the PH assumption was not met, and to avoid imposing the strong assumption of constant risk reduction over time, NNT was derived from estimated survival probabilities at prespecified time points using the approach proposed by Altman.^41^ The standard error of the Kaplan-Meier survival probability was calculated using Greenwood’s formula,^43^ which considers both the number of patients at risk and the number of events at each time point. By quantifying treatment benefit on an absolute scale, NNT facilitates interpretation of modeled cost differences in terms of the number of patients treated to avert one additional event, with lower NNT values indicating greater clinical impact.

Although combination therapies may offer substantial clinical benefits, they often face challenges in demonstrating cost-effectiveness, even when the add-on component is hypothetically priced at zero.^27^^,^^44^^,^^45^^,^^46^ To address this “not cost-effective at zero price” paradox, we applied a value attribution framework to allocate economic value within the EC + mFOLFOX6 regimen.^27^ Value attribution followed Briggs’ framework of imperfect information with balance or imbalance of market power, which is independent of drug prices and relies solely on incremental QALYs for the allocation of value.^27^ The balanced market power scenario is described as assuming symmetric bargaining between regimen components, whereas the imbalanced market power scenario allows a greater share of incremental value to accrue to the backbone component, reflecting asymmetric bargaining power or informational advantage. This approach provides a structured method for allocating economic value between the components of a combination regimen under alternative assumptions regarding information asymmetry and market power. The health outcomes represent the incremental QALYs gained relative to the SOC comparator, and the monetary value of monotherapy was derived by multiplying the respective incremental QALYs by the WTP threshold of $150000. Within the value attribution framework, EC was defined as the backbone therapy because it represents the established targeted regimen and the reference comparator for BRAF V600E-mutant mCRC. The addition of mFOLFOX6 substantially increased incremental QALYs but at a disproportionately high cost, a profile consistent with an add-on component in economic evaluation. However, the value of encorafenib and cetuximab could not be separately attributed, as there is no robust clinical evidence supporting their use as monotherapies in mCRC.

### Quantification and statistical analysis

This study was a model-based economic evaluation using reconstructed survival data derived from published Kaplan–Meier curves of the BREAKWATER trial. Survival data were digitally extracted and reconstructed into pseudo–individual patient data, which were used to fit parametric and flexible spline-based survival models for progression-free and overall survival. The proportional hazards assumption was assessed using log cumulative hazard plots, and non-proportional hazards were addressed by fitting models separately for each treatment arm. Model outcomes included life-years, quality-adjusted life-years, costs, and incremental cost-effectiveness measures, with uncertainty explored through deterministic sensitivity analyses, probabilistic sensitivity analyses, and scenario analyses. All analyses were conducted using R (version 4.4.2) and Microsoft Excel.
