# Supplementary material for: Cost-effectiveness of first-line encorafenib plus cetuximab with mFOLFOX6 in BRAF V600E-mutant metastatic colorectal cancer
Source: iScience. 2026 Feb 10;29(3):114977. doi: 10.1016/j.isci.2026.114977 (PMC12964229; doi:10.1016/j.isci.2026.114977)
Supplement: Document S1. Figures S1–S12 and Tables S1–S8 [file mmc1.pdf]

iScience, Volume *29*

Supplemental information

Cost-effectiveness of first-line encorafenib plus cetuximab with mFOLFOX6 in BRAF V600E-mutant metastatic colorectal cancer

Yamin Shu, Fenghao Shi, Jinlin Xiong, Jienan Zheng, Yiling Ding, Wenting Zhang, Pingping Xu, and Qilin Zhang

Supplemental Materials Content

Figure S1. Two-way sensitivity analysis of encorafenib and cetuximab costs
Figure S2. Decision tree structure integrated with a three-state partitioned survival model
Figure S3. Kaplan-Meier PFS reconstruction for EC+mFOLFOX6, EC, and SOC
Figure S4. Kaplan-Meier OS reconstruction for EC+mFOLFOX6, EC, and SOC
Figure S5. Fitted seven standard survival models compared with original Kaplan-Meier curves
Figure S6. Comparison between spline-fitted survival models and original Kaplan-Meier curves
Figure S7. Fit of AIC-weighted averaged survival models relative to Kaplan-Meier data
Figure S8. Fit of BIC-weighted averaged survival models relative to Kaplan-Meier data
Figure S9. Testing the proportional hazards assumption for PFS using log cumulative hazard plots
Figure S10. Testing the proportional hazards assumption for OS using log cumulative hazard plots
Figure S11. Model-based predictions of hazard functions
Figure S12. General population utility over time

Table S1. Summary of cost and outcome results in the PSA
Table S2. Scenario analysis results
Table S3. NNT to prevent one progression event at different time points across treatment comparisons
Table S4. NNT to prevent one death event at different time points across treatment comparison
Table S5. Evaluation of survival outcomes from original and reconstructed Kaplan-Meier curves
Table S6. Model-specific AIC and BIC values and associated median PFS and OS estimates
Table S7. Model-specific parameter estimates from survival analyses
Table S8. Variance-covariance matrix of model parameters was decomposed using the Cholesky method

Figure S1. Two-way sensitivity analysis of encorafenib and cetuximab costs

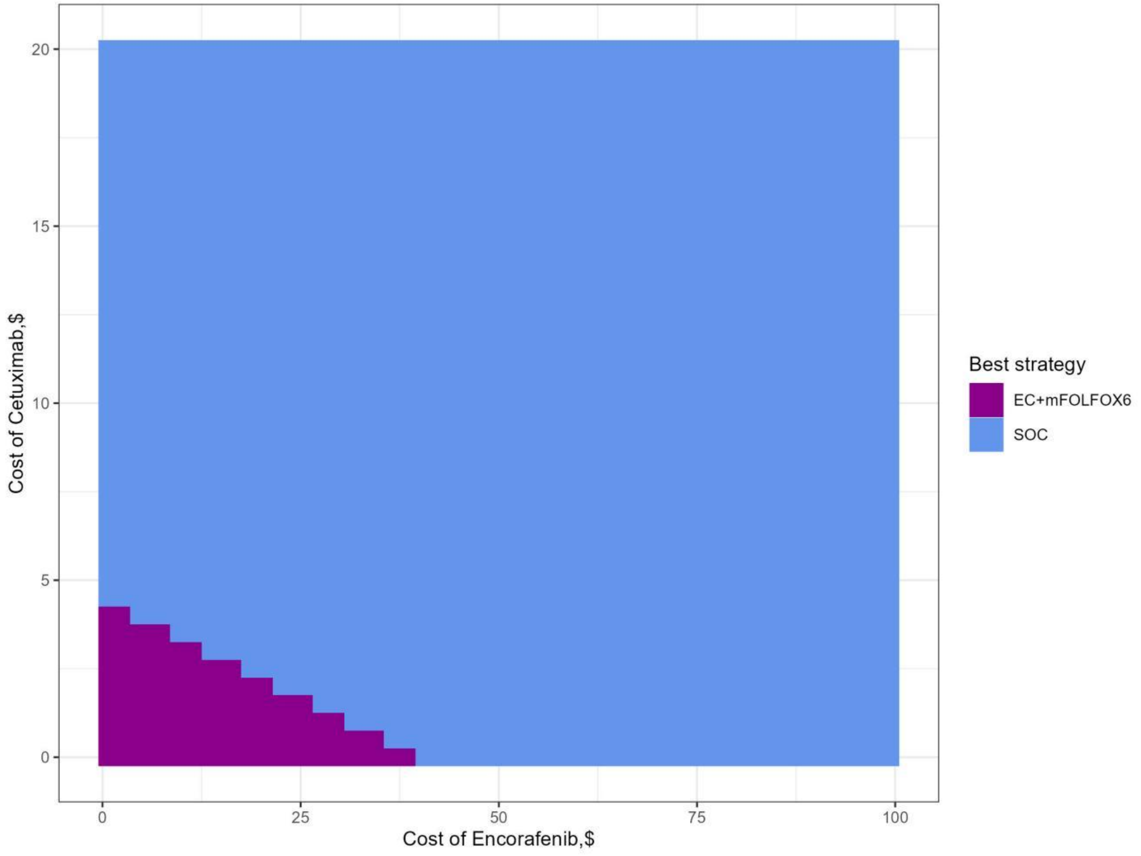

Figure S2. Decision tree structure integrated with a three-state partitioned survival model

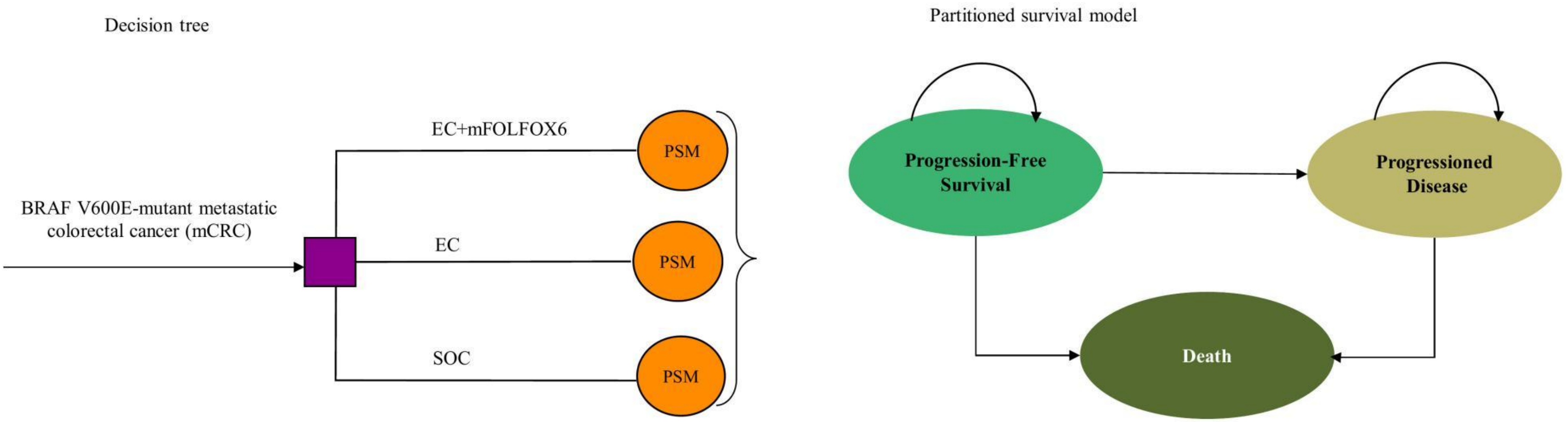

Figure S3. Kaplan-Meier PFS reconstruction for EC+mFOLFOX6, EC, and SOC

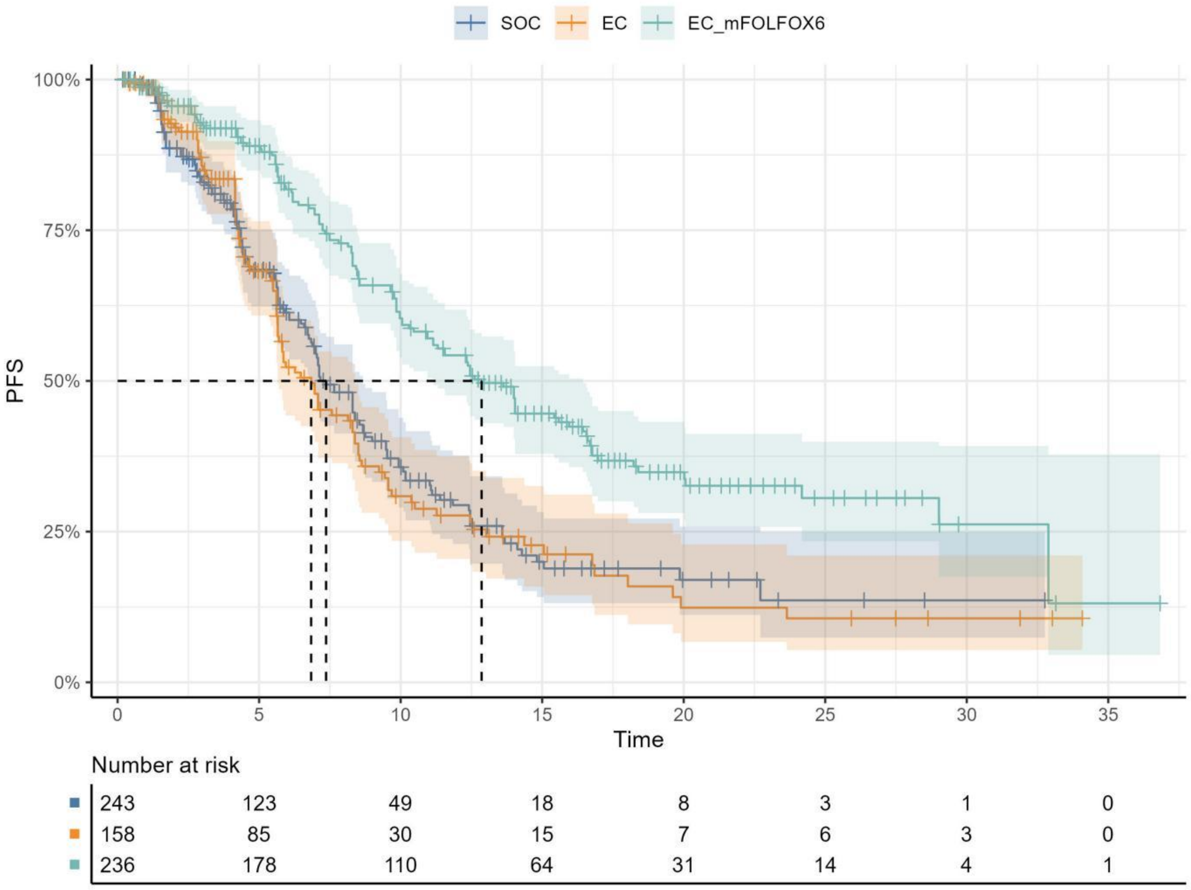

Figure S4. Kaplan-Meier OS reconstruction for EC+mFOLFOX6, EC, and SOC

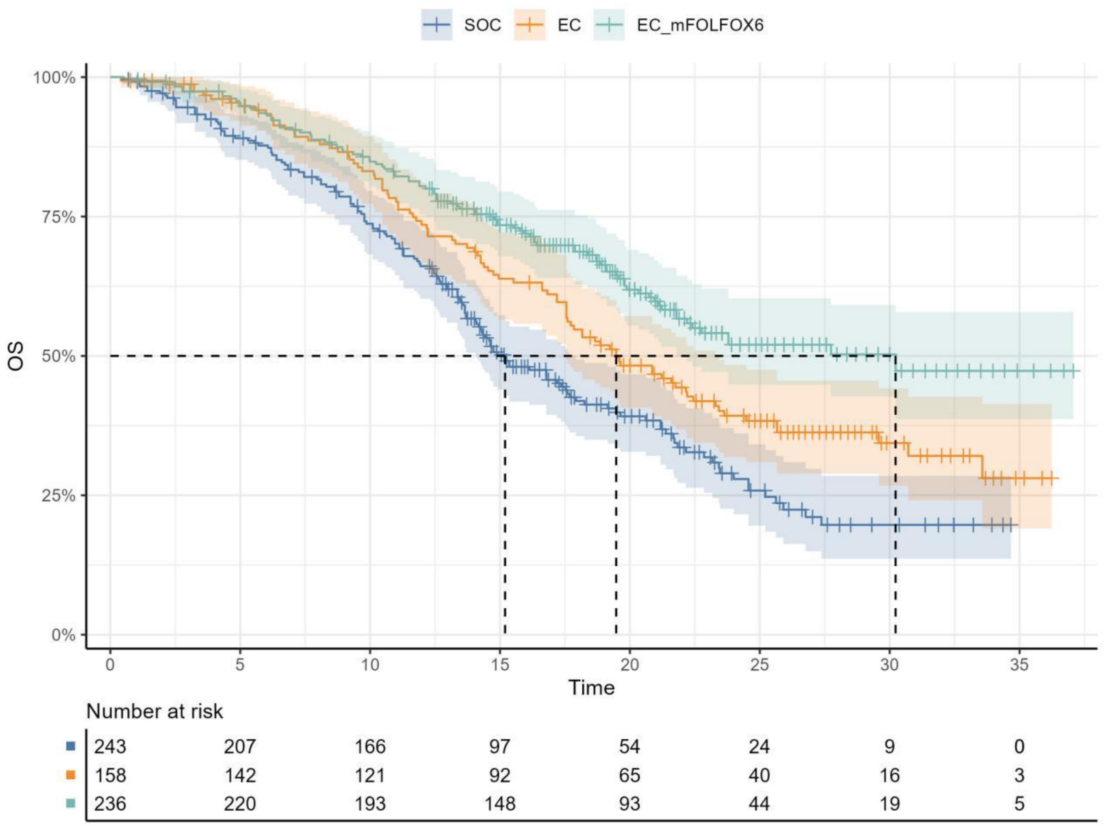

Figure S5. Fitted seven standard survival models compared with original Kaplan-Meier curves

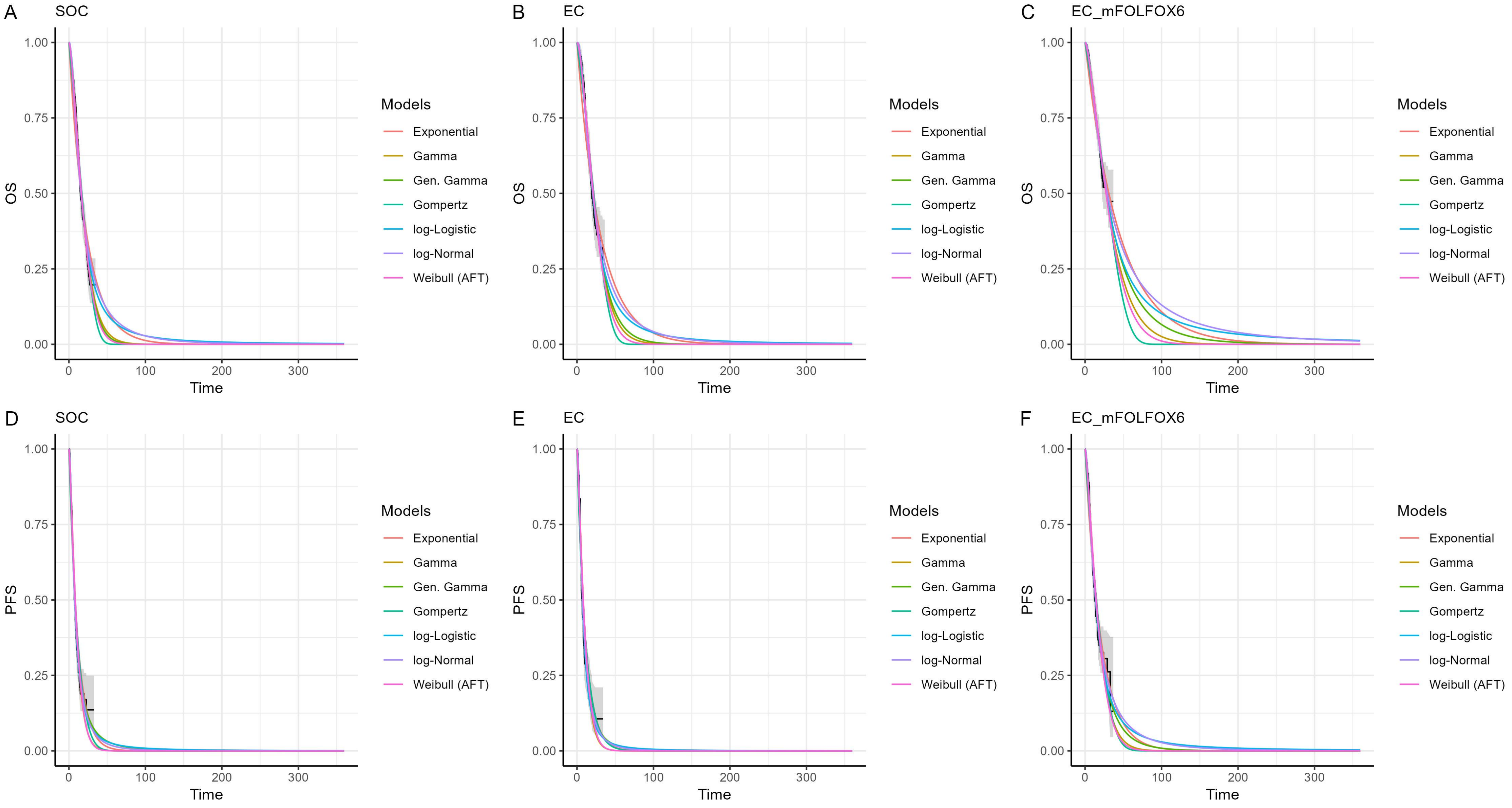

Figure S6. Comparison between spline-fitted survival models and original Kaplan-Meier curves

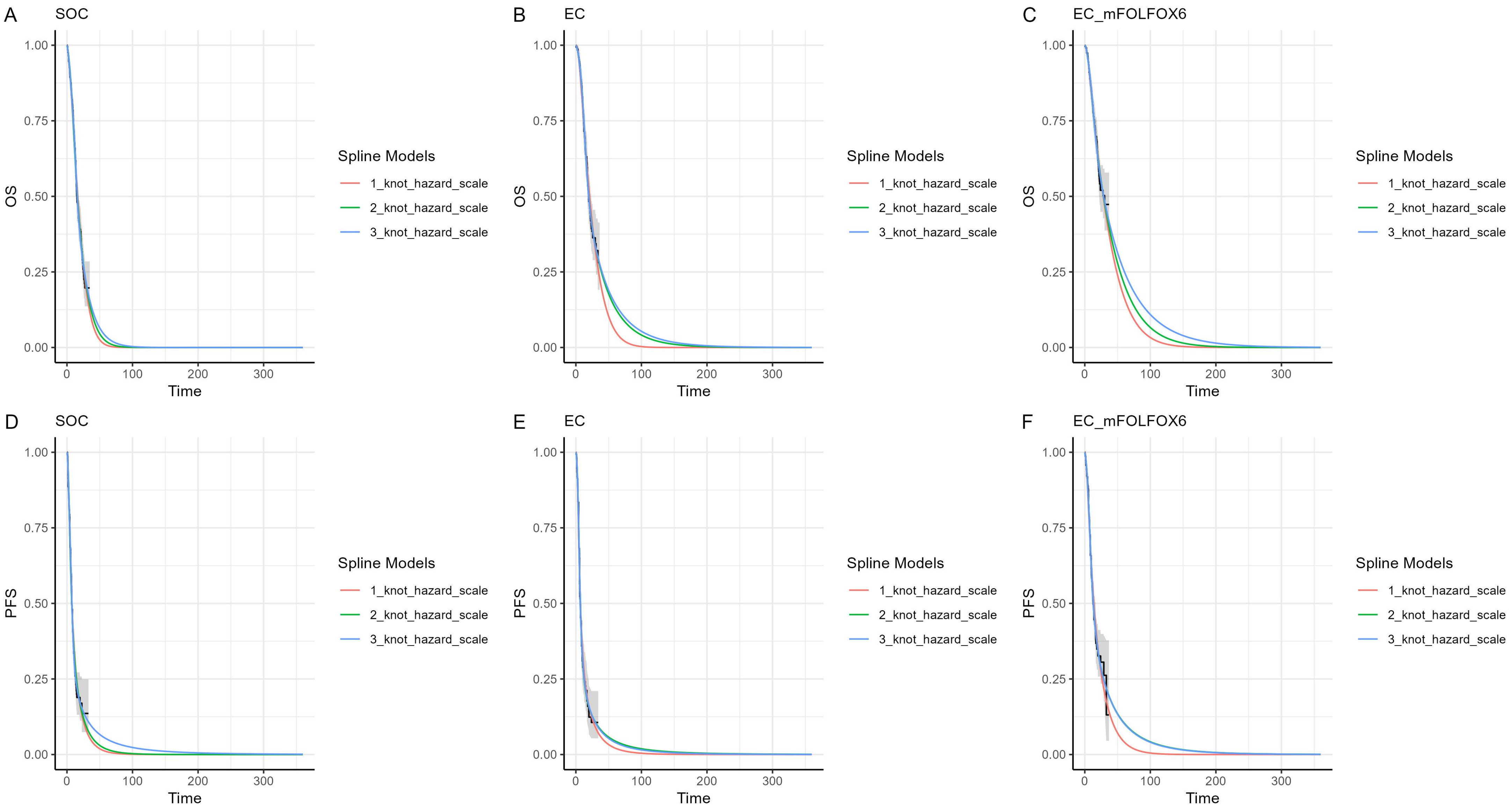

Figure S7. Fit of AIC-weighted averaged survival models relative to Kaplan-Meier data

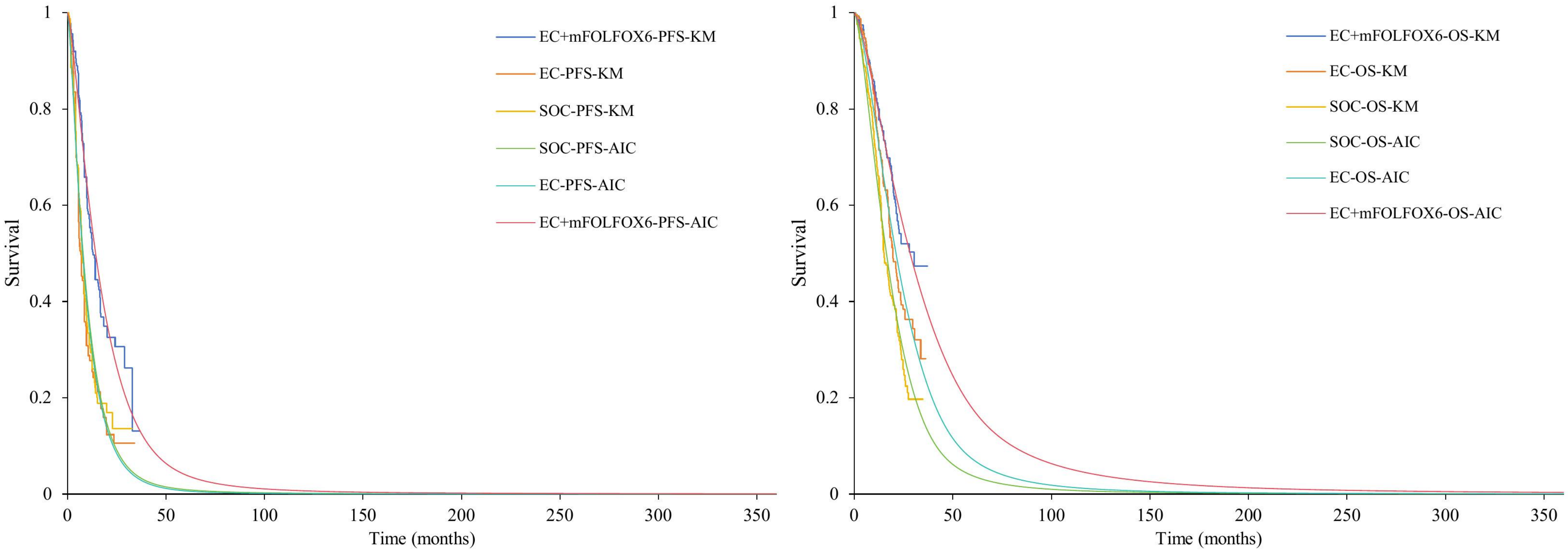

Figure S8. Fit of BIC-weighted averaged survival models relative to Kaplan-Meier data

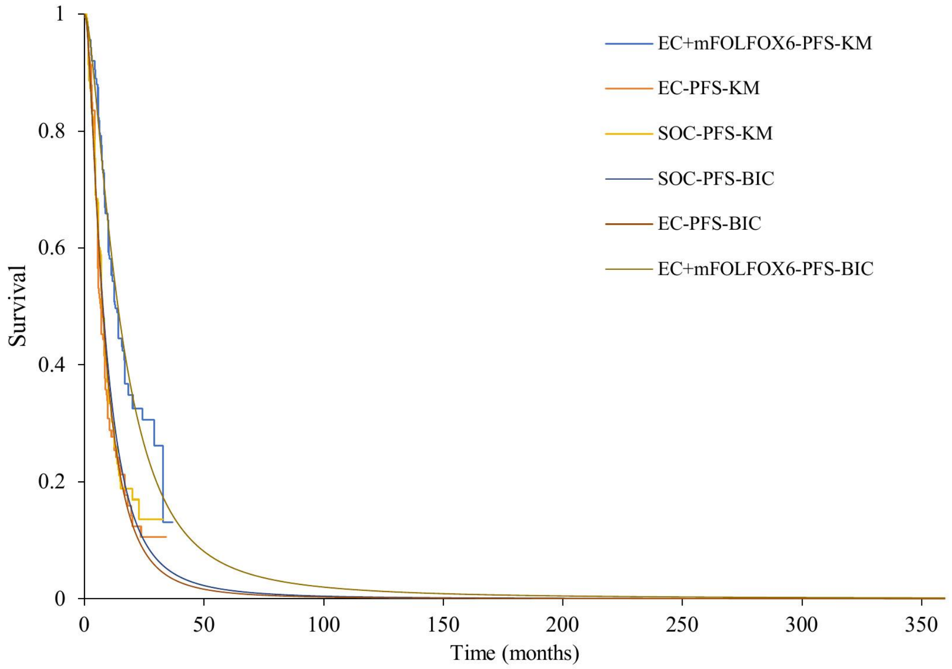

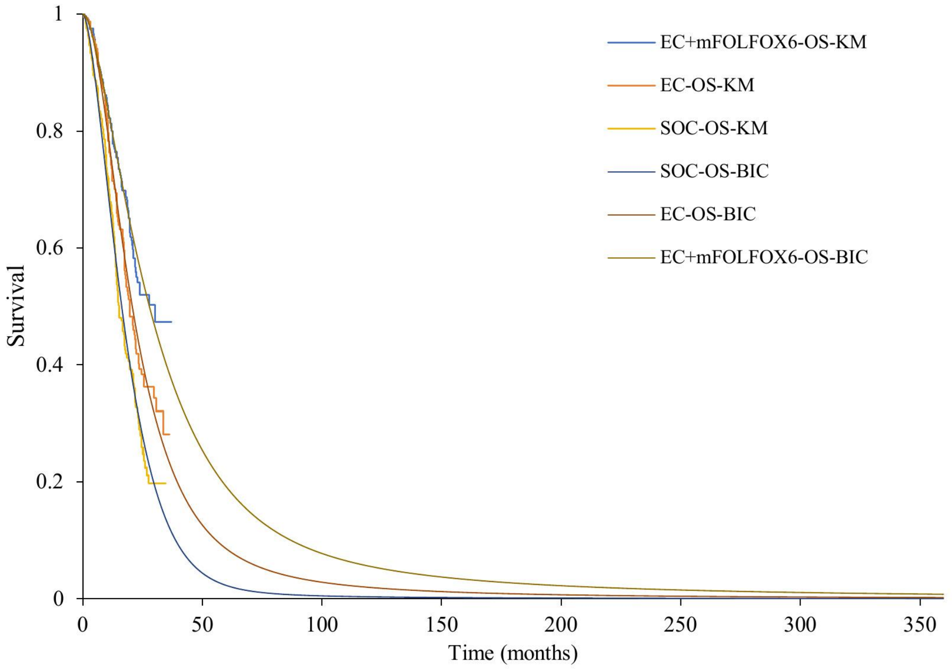

Figure S9. Testing the proportional hazards assumption for PFS using log cumulative hazard plots

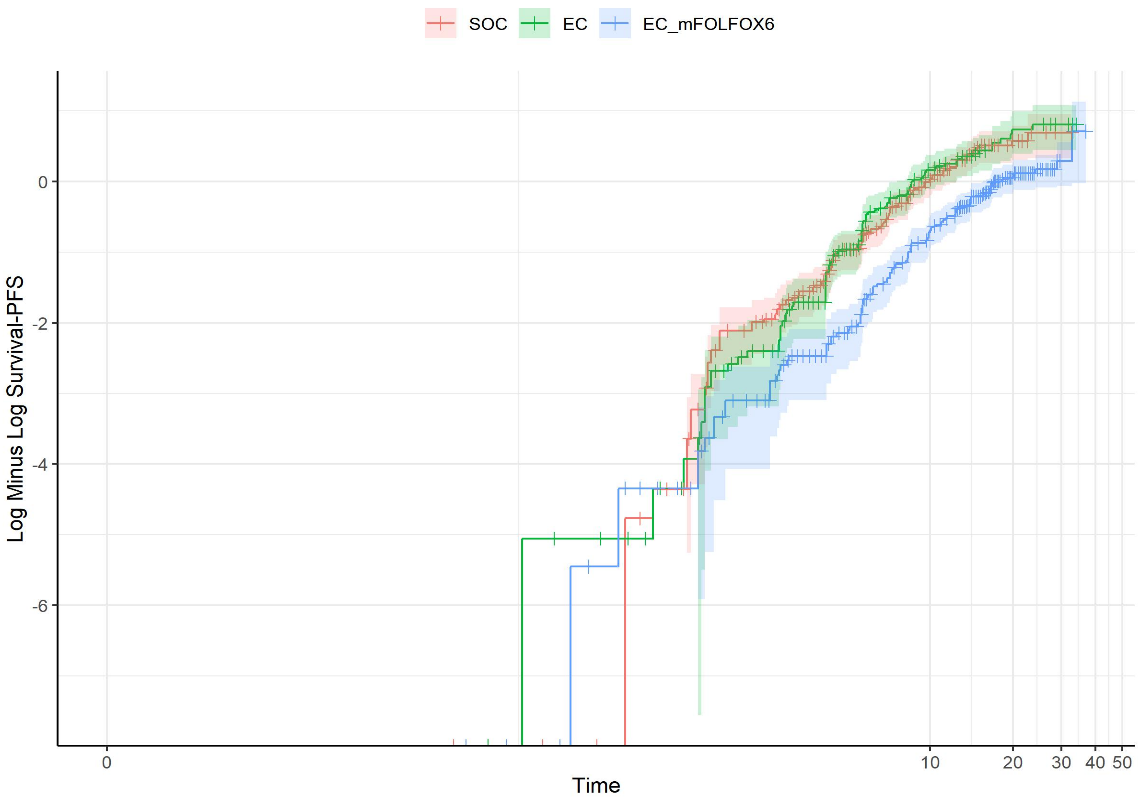

Figure S10. Testing the proportional hazards assumption for OS using log cumulative hazard plots

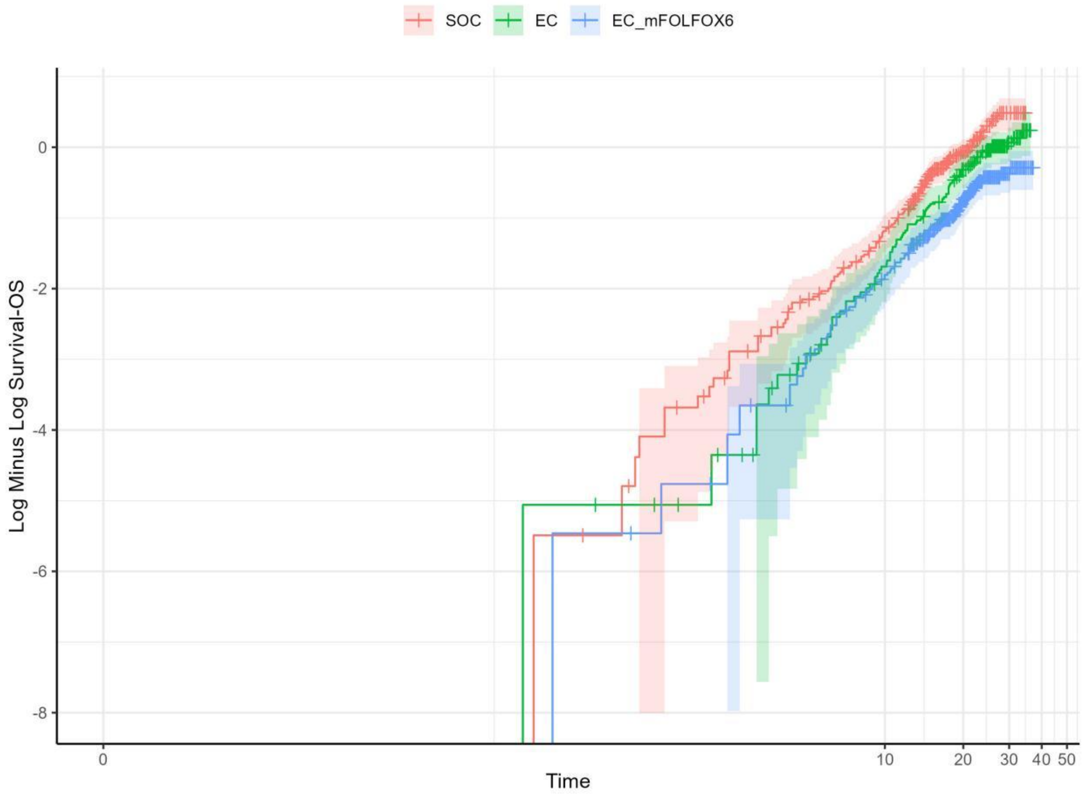

Figure S11. Model-based predictions of hazard functions

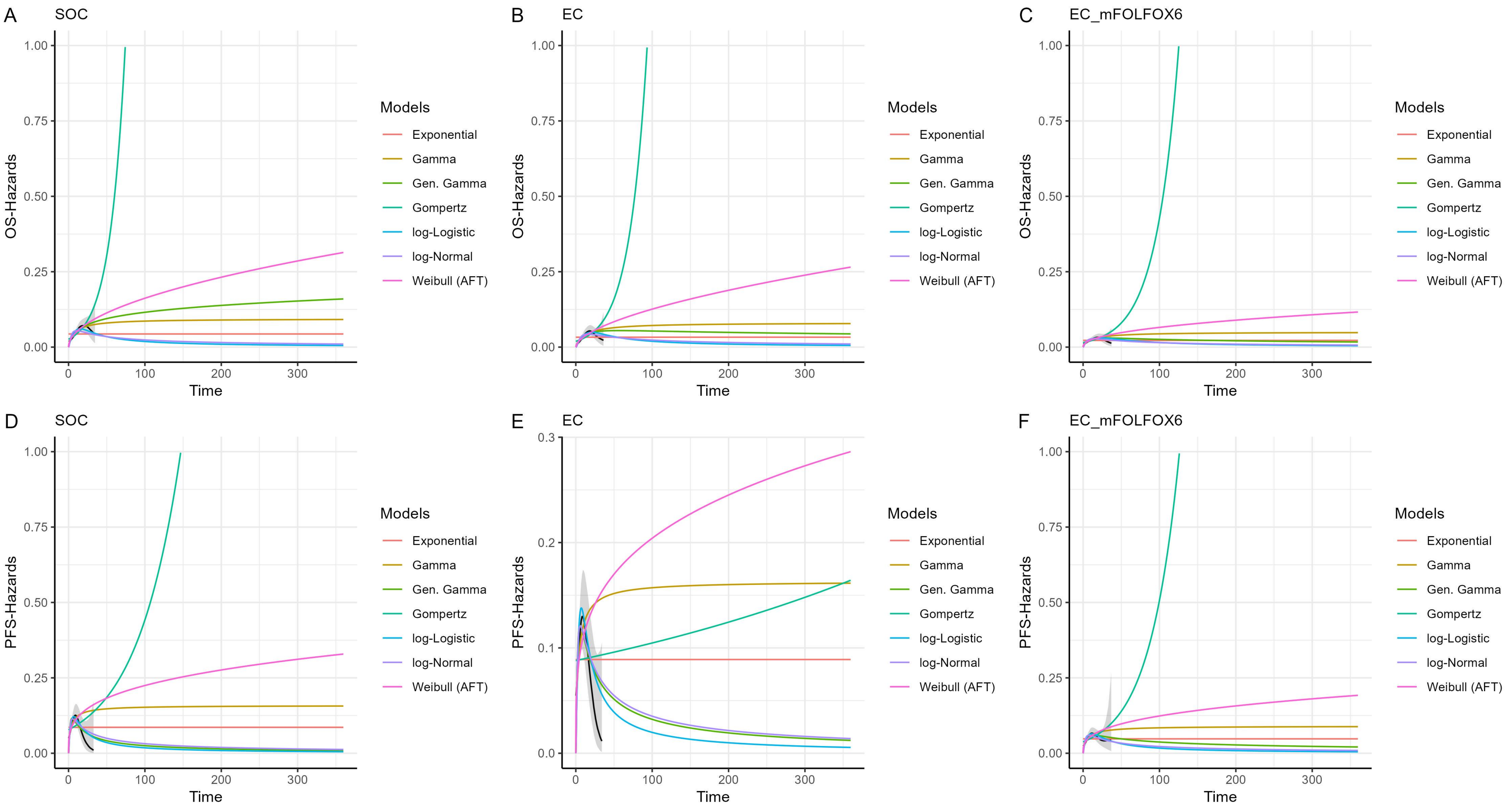

Figure S12. General population utility over time

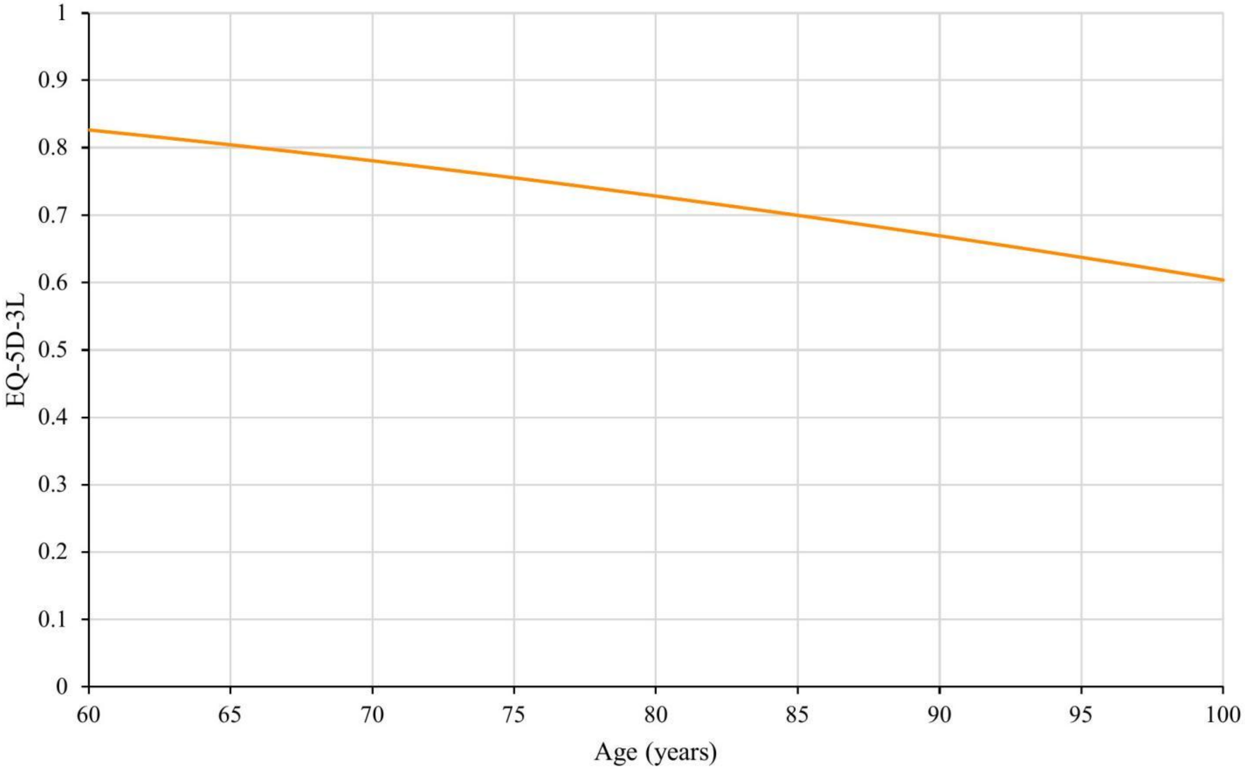

Table S1. Summary of cost and outcome results in the PSA

Variables	EC+mFOLFOX6	EC	SOC	Incremental		
				EC+mFOLFOX6 *vs.* EC	EC+mFOLFOX6 *vs.* SOC	EC *vs.* SOC
Total costs, mean (95% CI), $	883919 (718094, 1087598)	552403 (470878, 653996)	229544 (198714, 263573)	331516 (192772, 505470)	654375 (492850, 854772)	322859 (240111, 421956)
Drug acquisition	583023 (461594, 737166)	293263 (230264, 371123)	74184 (59965, 90628)	289760 (172045, 435375)	508838 (387824, 663090)	219079 (154122, 296206)
Drug administration	26061 (21128, 32105)	3112 (2476, 3934)	10157 (8506, 11935)	22948 (18135, 28886)	15903 (10973, 22039)	-7045 (-8888, -5339)
Subsequent treatment	220207 (114444, 361627)	212261 (145243, 298244)	95916 (62401, 134942)	7946 (-112263, 149509)	124291 (12186, 265620)	116345 (41788, 202391)
Monitoring	11851 (9754, 14435)	6454 (5308, 7836)	6766 (5707, 7924)	5397 (3208, 8002)	5085 (2953, 7567)	-312 (-1795, 1219)
Adverse event	8430 (7934, 8967)	1038 (962, 1117)	4915 (4553, 5311)	7392 (6916, 7909)	3515 (3045, 3992)	-3877 (-4254, -3526)
End of life	34348 (28678, 40047)	36275 (31649, 40993)	37606 (33479, 41880)	-1927 (-4801, -218)	-3259 (-7320, -850)	-1331 (-3359, -239)
Total LYs, mean (95% CI), year	3.183 (2.46, 4.131)	2.285 (1.887, 2.805)	1.574 (1.398, 1.776)	0.898 (0.202, 1.785)	1.609 (0.899, 2.528)	0.711 (0.291, 1.229)
PFS	1.751 (1.438, 2.149)	0.904 (0.738, 1.108)	0.95 (0.802, 1.11)	0.847 (0.496, 1.265)	0.801 (0.462, 1.206)	-0.046 (-0.275, 0.194)
PD	1.432 (0.752, 2.319)	1.381 (0.958, 1.922)	0.624 (0.412, 0.86)	0.051 (-0.727, 0.964)	0.808 (0.079, 1.695)	0.757 (0.277, 1.315)
Total QALYs, mean (95% CI), QALY	2.445 (1.907, 3.14)	1.729 (1.435, 2.12)	1.209 (1.075, 1.36)	0.716 (0.198, 1.367)	1.236 (0.707, 1.909)	0.52 (0.21, 0.906)
PFS	1.401 (1.143, 1.723)	0.722 (0.588, 0.888)	0.759 (0.638, 0.891)	0.679 (0.399, 1.015)	0.642 (0.368, 0.968)	-0.037 (-0.221, 0.155)
PD	1.048 (0.549, 1.702)	1.01 (0.699, 1.409)	0.456 (0.301, 0.631)	0.038 (-0.538, 0.706)	0.592 (0.058, 1.241)	0.554 (0.205, 0.965)
Adverse Events Disutilities	0.00116 (0.0011, 0.00123)	0.0002 (0.00018, 0.00022)	0.00053 (0.0005, 0.00056)	0.00096 (0.0009, 0.00103)	0.00063 (0.00058, 0.00069)	-0.00033 (-0.00036, -0.0003)
Time to death Disutilities	0.00258 (0.00173, 0.00356)	0.00296 (0.00179, 0.00424)	0.00516 (0.00396, 0.00641)	-0.00037 (-0.00193, 0.00111)	-0.00258 (-0.00411, -0.00107)	-0.00221 (-0.00381, -0.00046)
ICER, mean (95% CI), $/LY				421601 (247671, 1027119)	422837 (313948, 597557)	495742 (319286, 882736)
ICER, mean (95% CI), $/QALY				507856 (326859, 1046336)	548319 (412674, 758836)	680325 (433221, 1218093)
INHB, mean (95% CI), QALY				-1.494 (-2.179, -0.956)	-3.127 (-3.964, -2.44)	-1.633 (-2.039, -1.285)
INMB, mean (95% CI), $				-224101 (-326874, -143438)	-468998 (-594673, -365993)	-244896 (-305809, -192800)

Table S2. Scenario analysis results

Scenario	EC+mFOLFOX6	EC	SOC	Incremental		
				EC+mFOLFOX6 *vs.* EC	EC+mFOLFOX6 *vs.* SOC	EC *vs.* SOC
Base case						
Total costs, $	922029	560796	229219	361234	692810	331576
Total LYs, year	3.368	2.336	1.572	1.032	1.797	0.765
Total QALYs, QALY	2.584	1.767	1.208	0.817	1.376	0.559
Incremental costs per LY, $				350072	385586	433496
Incremental costs per QALY, $				442033	503391	593077
Scenario 1: Spline model						
Total costs, $	779296	524791	222659	254504	556637	302133
Total LYs, year	2.727	2.013	1.525	0.714	1.202	0.488
Total QALYs, QALY	2.099	1.535	1.173	0.564	0.926	0.362
Incremental costs per LY, $				356329	463074	619367
Incremental costs per QALY, $				451450	601394	835013
Scenario 2: AIC model						
Total costs, $	819596	526212	246901	293384	572695	279310
Total LYs, year	2.968	2.125	1.680	0.843	1.288	0.445
Total QALYs, QALY	2.276	1.610	1.285	0.666	0.991	0.325
Incremental costs per LY, $				347997	444638	627754
Incremental costs per QALY, $				440725	578029	859187
Scenario 3: BIC model						
Total costs, $	865938	543835	234088	322103	631850	309747
Total LYs, year	3.134	2.232	1.609	0.902	1.525	0.623
Total QALYs, QALY	2.406	1.690	1.236	0.715	1.169	0.454
Incremental costs per LY, $				357122	414232	496859
Incremental costs per QALY, $				450276	540341	682250
Scenario 4: Only progression-based utility						
Total costs, $	922029	560796	229219	361234	692810	331576
Total LYs, year	3.368	2.336	1.572	1.032	1.797	0.765
Total QALYs, QALY	2.592	1.774	1.217	0.818	1.375	0.557
Incremental costs per LY, $				350072	385586	433496
Incremental costs per QALY, $				441681	504038	595654
Scenario 5: Excluded relative dose intensity						
Total costs, $	964205	582050	229219	382155	734986	352831
Total LYs, year	3.368	2.336	1.572	1.032	1.797	0.765
Total QALYs, QALY	2.584	1.767	1.208	0.817	1.376	0.559
Incremental costs per LY, $				370347	409059	461284
Incremental costs per QALY, $				467634	534036	631095

Table S3. NNT to prevent one progression event at different time points across treatment comparisons

PFS	ECF *vs.* SOC			ECF *vs.* EC			EC *vs.* SOC		
time (months)	NNT	NNT (95%CI)	risk	NNT	NNT (95%CI)	risk	NNT	NNT (95%CI)	risk
2	14.3	14.3 (8.4 to 48.1)	409	27.8	27.8 (11.5 to -67.3)	347	29.4	29.4 (10.7 to -38.6)	332
4	8.1	8.1 (5.3 to 16.8)	346	11.9	11.9 (6.5 to 72.6)	302	25.0	25 (8.3 to -24.5)	266
6	4.9	4.9 (3.4 to 8.4)	257	3.4	3.4 (2.5 to 5.2)	218	-11.1	-11.1 (48.4 to -5)	161
8	4.0	4 (2.9 to 6.6)	209	3.5	3.5 (2.5 to 5.6)	184	-26.3	-26.3 (13.1 to -6.6)	121
10	4.0	4 (2.9 to 6.8)	159	3.4	3.4 (2.5 to 5.4)	140	-20.8	-20.8 (15.5 to -6.2)	79
12	4.0	4 (2.9 to 6.8)	130	3.8	3.8 (2.7 to 6.4)	120	-58.8	-58.8 (10.7 to -7.8)	58
14	4.2	4.2 (2.9 to 7.2)	100	4.4	4.4 (3 to 8.4)	95	90.9	90.9 (8.4 to -10.3)	41
16	4.3	4.3 (3 to 7.4)	72	4.7	4.7 (3.1 to 9.7)	69	43.5	43.5 (7.7 to -12)	27
18	5.6	5.6 (3.6 to 12.9)	50	5.2	5.2 (3.3 to 12.4)	49	-83.3	-83.3 (10.6 to -8.4)	21
20	5.6	5.6 (3.5 to 13.2)	39	4.4	4.4 (3 to 8.5)	38	-21.7	-21.7 (17.2 to -6.7)	15
22	6.4	6.4 (3.8 to 19.4)	28	5.0	5 (3.2 to 10.6)	29	-21.7	-21.7 (17.2 to -6.7)	13
24	5.3	5.3 (3.3 to 12.9)	19	4.5	4.5 (3.1 to 8.7)	22	-33.3	-33.3 (12.5 to -7.2)	9
26	5.9	5.9 (3.5 to 18.4)	15	5.0	5 (3.2 to 11)	17	-33.3	-33.3 (12.5 to -7.2)	8
28	5.9	5.9 (3.5 to 18.4)	10	5.0	5 (3.2 to 11)	12	-33.3	-33.3 (12.5 to -7.2)	6
30	7.9	7.9 (3.8 to -126.1)	5	6.4	6.4 (3.5 to 35.9)	7	-33.3	-33.3 (12.5 to -7.2)	4

Table S4. NNT to prevent one death event at different time points across treatment comparisons

OS	ECF *vs.* SOC			ECF *vs.* EC			EC *vs.* SOC		
time (months)	NNT	NNT (95%CI)	risk	NNT	NNT (95%CI)	risk	NNT	NNT (95%CI)	risk
2	47.6	47.6 (22.1 to -312.9)	466	-500.0	-500 (66.5 to -52.5)	387	43.5	43.5 (21 to -659.6)	387
4	20.4	20.4 (11.3 to 101.4)	444	76.9	76.9 (20.1 to -42.1)	372	27.8	27.8 (12.3 to -105.7)	362
6	16.9	16.9 (9 to 150)	418	500.0	500 (19 to -20.6)	353	17.5	17.5 (8.7 to -1775.4)	339
8	14.1	14.1 (7.4 to 145.1)	390	500.0	500 (14.9 to -15.8)	334	14.5	14.5 (7.1 to -468.8)	316
10	9.0	9 (5.4 to 26.5)	359	58.8	58.8 (10.7 to -16.9)	314	10.6	10.6 (5.7 to 90.1)	287
12	7.0	7 (4.5 to 15.9)	329	14.5	14.5 (6.4 to -52.9)	289	13.5	13.5 (5.9 to -49.7)	254
14	5.1	5.1 (3.5 to 9)	277	14.3	14.3 (6.1 to -43.9)	262	7.9	7.9 (4.4 to 35.7)	217
16	4.2	4.2 (3 to 6.7)	225	11.5	11.5 (5.4 to -90.5)	230	6.6	6.6 (3.9 to 20.4)	177
18	3.7	3.7 (2.8 to 5.7)	185	7.1	7.1 (4.1 to 26.3)	199	7.8	7.8 (4.3 to 45.5)	142
20	4.4	4.4 (3.1 to 7.7)	147	7.4	7.4 (4.1 to 33.4)	158	11.0	11 (5.1 to -62.6)	119
22	4.3	4.3 (3 to 7.7)	110	8.1	8.1 (4.3 to 72.5)	124	9.3	9.3 (4.6 to -5436.8)	94
24	4.1	4.1 (2.9 to 7.4)	75	7.9	7.9 (4.2 to 69.5)	92	8.8	8.8 (4.5 to 206.5)	71
26	3.4	3.4 (2.5 to 5.3)	57	6.4	6.4 (3.7 to 22.7)	72	7.2	7.2 (4 to 34)	53
28	3.3	3.3 (2.4 to 5.1)	40	7.1	7.1 (3.9 to 41.9)	53	6.0	6 (3.6 to 17.9)	39
30	3.3	3.3 (2.4 to 5.1)	28	6.3	6.3 (3.6 to 24.9)	35	6.8	6.8 (3.8 to 29.3)	25

Table S5. Evaluation of survival outcomes from original and reconstructed Kaplan–Meier curves

Survival curve	group	records	events	median	0.95LCL	0.95UCL
	SOC	243	132	7.1	6.8	8.5
PFS in BREAKWATER	EC	158	/	6.8	5.7	8.3
	EC+mFOLFOX6	236	122	12.8	11.2	15.9
	SOC	243	148	15.1	13.7	17.7
OS in BREAKWATER	EC	158	/	19.5	17.6	22.5
	EC+mFOLFOX6	236	94	30.3	21.7	NA
	SOC	243	138	7.4	6.8	8.7
PFS in Replicated Kaplan-Meier	EC	158	100	6.8	5.7	8.4
	EC+mFOLFOX6	236	123	12.9	11.2	16.4
	SOC	243	149	15.2	14.1	17.7
OS in Replicated Kaplan-Meier	EC	158	92	19.5	17.6	23.4
	EC+mFOLFOX6	236	94	30.2	21.8	NA

Table S6. Model-specific AIC and BIC values and associated median PFS and OS estimates

Strategies	Models	PFS						OS				
		distribution	AIC	BIC	median_est	median_lcl	median_ucl	AIC	BIC	median_est	median_lcl	median_ucl
SOC	Classic models	exp	955.14	958.63	8.1	6.8	9.5	1233.46	1236.95	15.9	13.5	18.6
		gamma	936.95	943.94	8.2	7.2	9.3	1204.85	1211.84	16.2	14.5	18.2
		gengamma	926.53	937.01	7.5	6.4	8.7	1206.59	1217.07	16.3	14.6	18.2
		gompertz	955.88	962.87	8.3	6.9	9.7	1215.10	1222.09	17.0	15.1	18.8
		llogis	926.87	933.86	7.6	6.6	8.6	1206.62	1213.61	15.9	14.1	18.1
		lnorm	924.85	931.83	7.6	6.6	8.7	1217.45	1224.44	15.8	13.8	18.2
		weibull	942.79	949.77	8.4	7.3	9.5	1204.89	1211.88	16.4	14.7	18.4
	Spline models	1_knot_hazard_scale	930.09	940.57	7.4	6.4	8.6	1206.85	1217.33	16.4	14.6	18.4
		2_knot_hazard_scale	931.13	945.10	7.3	6.4	8.5	1206.85	1220.82	15.9	14.2	17.8
		3_knot_hazard_scale	925.03	942.50	7.5	6.7	8.5	1205.24	1222.70	15.7	14.3	17.6
EC	Classic models	exp	685.85	688.92	7.8	6.4	9.5	814.40	817.46	21.1	17.0	25.8
		gamma	672.66	678.79	8.2	7.0	9.4	792.53	798.65	21.1	18.2	24.0
		gengamma	662.01	671.20	7.4	6.3	8.7	794.21	803.39	20.9	18.4	24.2
		gompertz	687.84	693.97	7.8	6.3	9.6	804.60	810.72	21.9	18.8	24.9
		llogis	656.72	662.85	7.2	6.3	8.4	790.06	796.19	20.5	17.8	23.7
		lnorm	660.07	666.20	7.4	6.3	8.7	796.58	802.70	20.7	17.8	24.4
		weibull	678.83	684.96	8.3	7.0	9.7	794.49	800.62	21.4	18.6	24.4
	Spline models	1_knot_hazard_scale	658.32	667.51	6.9	5.9	8.3	794.49	803.68	20.9	17.7	24.1
		2_knot_hazard_scale	655.43	667.68	6.8	6.0	7.9	791.93	804.18	19.7	16.7	23.3
		3_knot_hazard_scale	657.17	672.48	6.7	5.8	7.9	793.47	808.78	19.6	16.8	23.3
EC+mFOLFOX6	Classic models	exp	996.32	999.79	14.5	12.2	17.2	905.27	908.73	31.1	25.7	38.4
		gamma	980.95	987.88	14.4	12.6	16.2	891.60	898.53	27.5	23.4	31.9
		gengamma	980.15	990.54	14.0	12.2	16.3	892.73	903.12	28.0	23.6	33.8
		gompertz	994.24	1001.16	14.9	12.9	17.1	900.58	907.51	27.9	24.3	32.8
		llogis	976.16	983.09	13.7	11.8	15.7	890.15	897.08	27.7	23.4	32.8
		lnorm	979.93	986.86	13.8	12.0	16.3	892.31	899.24	29.2	23.9	35.2
		weibull	983.89	990.82	14.6	12.8	16.6	892.97	899.90	27.5	24.0	32.0
	Spline models	1_knot_hazard_scale	979.82	990.21	13.8	11.7	16.2	893.19	903.58	28.1	24.1	34.8
		2_knot_hazard_scale	976.39	990.25	13.0	11.2	15.4	894.40	908.26	28.6	23.7	38.4
		3_knot_hazard_scale	978.43	995.75	13.0	11.3	15.6	895.85	913.17	28.8	23.4	44.9

Table S7. Model-specific parameter estimates from survival analyses

Strategies	Models	distribution	parameter	PFS				OS			
				est	L95%	U95%	se	est	L95%	U95%	se
SOC	Classic models	exp	rate	-2.45341	-2.62025	-2.28656	0.08513	-3.13241	-3.29297	-2.97184	0.08192
		gamma	shape	0.48065	0.28228	0.67902	0.10121	0.60967	0.40807	0.81127	0.10286
		gamma	rate	-1.84329	-2.11582	-1.57077	0.13905	-2.36584	-2.63295	-2.09874	0.13628
		gengamma	mu	1.96115	1.69209	2.23021	0.13728	3.00675	2.84019	3.17330	0.08498
		gengamma	sigma	-0.02870	-0.16652	0.10912	0.07032	-0.35967	-0.59605	-0.12328	0.12061
		gengamma	Q	-0.15996	-0.72681	0.40689	0.28921	0.86022	0.37503	1.34541	0.24755
		gompertz	shape	0.01738	-0.01230	0.04706	0.01514	0.04905	0.02851	0.06959	0.01048
		gompertz	rate	-2.55411	-2.79950	-2.30871	0.12520	-3.64439	-3.93586	-3.35291	0.14871
		llogis	shape	0.60346	0.46795	0.73897	0.06914	0.65652	0.51948	0.79356	0.06992
		llogis	scale	2.02230	1.88604	2.15856	0.06952	2.76831	2.64786	2.88876	0.06146
		lnorm	meanlog	2.02492	1.88357	2.16628	0.07212	2.75710	2.61960	2.89459	0.07015
		lnorm	sdlog	-0.05004	-0.17003	0.06996	0.06122	-0.03474	-0.15374	0.08426	0.06072
		weibull	shape	0.26074	0.13487	0.38660	0.06422	0.41599	0.28138	0.55060	0.06868
		weibull	scale	2.40844	2.27948	2.53739	0.06579	3.04199	2.93478	3.14920	0.05470
	Spline models	1_knot	gamma0	-3.98383	-4.68156	-3.28610	0.35599	-4.68775	-5.69538	-3.68012	0.51411
		1_knot	gamma1	2.18598	1.63841	2.73356	0.27938	1.57969	0.88819	2.27119	0.35281
		1_knot	gamma2	0.16114	0.07276	0.24951	0.04509	0.01019	-0.09493	0.11531	0.05363
		2_knot	gamma0	-3.89246	-4.60852	-3.17641	0.36534	-4.55074	-5.51008	-3.59140	0.48947
		2_knot	gamma1	1.98465	1.25114	2.71816	0.37425	1.25277	0.48356	2.02197	0.39246
		2_knot	gamma2	-0.16174	-0.71186	0.38838	0.28068	-0.48616	-1.16466	0.19234	0.34618
		2_knot	gamma3	0.34064	-0.23467	0.91594	0.29353	0.70755	-0.26516	1.68026	0.49629
		3_knot	gamma0	-4.05932	-4.84808	-3.27056	0.40244	-4.63167	-5.66225	-3.60109	0.52582
		3_knot	gamma1	2.71359	1.68206	3.74512	0.52630	1.65082	0.66944	2.63219	0.50071
		3_knot	gamma2	1.21046	0.29226	2.12866	0.46848	0.79787	-0.23240	1.82814	0.52566
		3_knot	gamma3	-2.74042	-4.63454	-0.84631	0.96640	-3.89274	-7.63322	-0.15225	1.90845
		3_knot	gamma4	2.12199	0.73501	3.50898	0.70766	3.84598	0.44087	7.25110	1.73734
EC	Classic models	exp	rate	-2.41927	-2.61527	-2.22327	0.10000	-3.41520	-3.61954	-3.21086	0.10426
		gamma	shape	0.50029	0.26319	0.73740	0.12097	0.70447	0.44215	0.96679	0.13384
		gamma	rate	-1.81254	-2.12700	-1.49807	0.16044	-2.51612	-2.86031	-2.17193	0.17561
		gengamma	mu	1.98172	1.74087	2.22257	0.12289	3.18201	2.98186	3.38217	0.10212
		gengamma	sigma	-0.09583	-0.24786	0.05620	0.07757	-0.28653	-0.54184	-0.03122	0.13026
		gengamma	Q	-0.05720	-0.51757	0.40318	0.23489	0.54591	0.01052	1.08130	0.27316
		gompertz	shape	0.00173	-0.02825	0.03171	0.01530	0.04229	0.01871	0.06587	0.01203
		gompertz	rate	-2.43030	-2.70550	-2.15510	0.14041	-3.95338	-4.34653	-3.56024	0.20059
		llogis	shape	0.68934	0.52808	0.85060	0.08228	0.70457	0.53129	0.87784	0.08841
		llogis	scale	1.97733	1.82703	2.12763	0.07668	3.01911	2.87412	3.16409	0.07397
		lnorm	meanlog	2.00396	1.84422	2.16370	0.08150	3.03166	2.86774	3.19558	0.08363
		lnorm	sdlog	-0.10303	-0.24351	0.03745	0.07167	-0.09245	-0.24609	0.06120	0.07839
		weibull	shape	0.23449	0.09159	0.37740	0.07291	0.45853	0.28366	0.63340	0.08922
		weibull	scale	2.40504	2.24999	2.56010	0.07911	3.29358	3.16138	3.42577	0.06745
	Spline model	1_knot	gamma0	-4.62902	-5.62096	-3.63707	0.50610	-6.43470	-8.52243	-4.34698	1.06519
		1_knot	gamma1	3.23719	2.26588	4.20850	0.49558	2.48168	1.11063	3.85272	0.69952
		1_knot	gamma2	0.20868	0.11354	0.30382	0.04854	0.12305	-0.05616	0.30225	0.09143
		2_knot	gamma0	-4.11315	-5.05261	-3.17370	0.47932	-5.40585	-7.26687	-3.54482	0.94952
		2_knot	gamma1	1.86996	0.53226	3.20766	0.68251	1.26495	-0.18038	2.71028	0.73743
		2_knot	gamma2	-0.63765	-1.24823	-0.02707	0.31153	-0.97164	-1.86764	-0.07564	0.45715
		2_knot	gamma3	0.87834	0.23318	1.52351	0.32917	1.54199	0.23837	2.84562	0.66512
		3_knot	gamma0	-4.14297	-5.07664	-3.20931	0.47637	-5.39937	-7.27931	-3.51943	0.95917
		3_knot	gamma1	1.68452	0.25274	3.11631	0.73052	1.26880	-0.32006	2.85766	0.81066
		3_knot	gamma2	-1.48004	-3.99360	1.03352	1.28245	-0.44733	-2.50808	1.61343	1.05142
		3_knot	gamma3	1.84177	-2.36811	6.05165	2.14794	-0.77636	-6.41218	4.85947	2.87547
		3_knot	gamma4	-0.17544	-2.17739	1.82651	1.02142	1.93162	-2.59973	6.46297	2.31196
EC+mFOLFOX6	Classic models	exp	rate	-3.04196	-3.21868	-2.86523	0.09017	-3.80460	-4.00676	-3.60245	0.10314
		gamma	shape	0.47514	0.26296	0.68733	0.10826	0.52454	0.27820	0.77087	0.12568
		gamma	rate	-2.40982	-2.70994	-2.10969	0.15313	-2.99909	-3.37911	-2.61906	0.19389
		gengamma	mu	2.74925	2.52192	2.97658	0.11599	3.47015	3.24601	3.69428	0.11436
		gengamma	sigma	-0.07317	-0.28224	0.13591	0.10667	-0.09255	-0.44279	0.25770	0.17870
		gengamma	Q	0.35385	-0.15709	0.86479	0.26069	0.43921	-0.23674	1.11516	0.34488
		gompertz	shape	0.02589	0.00148	0.05031	0.01246	0.03350	0.00868	0.05831	0.01266
		gompertz	rate	-3.26819	-3.55756	-2.97882	0.14764	-4.19959	-4.57718	-3.82201	0.19265
		llogis	shape	0.56027	0.41393	0.70662	0.07467	0.52871	0.35246	0.70495	0.08992
		llogis	scale	2.61476	2.46894	2.76059	0.07440	3.32042	3.15200	3.48885	0.08593
		lnorm	meanlog	2.62704	2.46686	2.78722	0.08173	3.37467	3.17832	3.57102	0.10018
		lnorm	sdlog	0.03026	-0.09958	0.16009	0.06625	0.09006	-0.06759	0.24771	0.08043
		weibull	shape	0.29559	0.15311	0.43807	0.07270	0.37127	0.19303	0.54951	0.09094
		weibull	scale	2.95382	2.81923	3.08840	0.06867	3.56556	3.40020	3.73093	0.08437
	Spline model	1_knot	gamma0	-4.90807	-5.95145	-3.86469	0.53235	-6.05966	-7.73032	-4.38901	0.85239
		1_knot	gamma1	2.19713	1.42148	2.97278	0.39575	2.10561	1.03882	3.17240	0.54429
		1_knot	gamma2	0.10176	0.01578	0.18775	0.04387	0.10109	-0.05401	0.25618	0.07913
		2_knot	gamma0	-4.46425	-5.41777	-3.51073	0.48650	-5.71433	-7.44677	-3.98189	0.88391
		2_knot	gamma1	1.40606	0.53699	2.27512	0.44341	1.69096	0.34449	3.03742	0.68699
		2_knot	gamma2	-0.56495	-1.06045	-0.06945	0.25281	-0.32354	-1.13505	0.48796	0.41404
		2_knot	gamma3	0.75980	0.18392	1.33568	0.29382	0.59226	-0.58174	1.76627	0.59899
		3_knot	gamma0	-4.46976	-5.41882	-3.52071	0.48422	-5.79601	-7.64491	-3.94710	0.94334
		3_knot	gamma1	1.36595	0.43185	2.30004	0.47659	1.83053	0.21464	3.44641	0.82445
		3_knot	gamma2	-0.59256	-2.05973	0.87462	0.74857	0.18635	-1.30786	1.68055	0.76236
		3_knot	gamma3	0.44357	-2.41970	3.30685	1.46088	-1.05879	-4.76712	2.64954	1.89204
		3_knot	gamma4	0.33586	-1.37443	2.04614	0.87261	1.49486	-1.89028	4.87999	1.72714

Table S8. Variance-covariance matrix of model parameters was decomposed using the Cholesky method

KM curves	strategies	distribution	parameter	est	Variance-Covariance matrix		Cholesky Decomposition	
PFS	SOC	lnorm	meanlog	2.02492482	0.005201451	0.0012638	0.0721211	0
		lnorm	sdlog	-0.05003595	0.001263751	0.0037481	0.0175226	0.0586602
	EC	llogis	shape	0.68933791	0.006769475	-0.001111	0.0822768	0
		llogis	scale	1.97733326	-0.001110687	0.0058805	-0.013499	0.0754871
	EC+mFOLFOX6	llogis	shape	0.56027404	0.005574977	-0.00132	0.0746658	0
		llogis	scale	2.61476299	-0.001320345	0.0055355	-0.017683	0.0722693
OS	SOC	gamma	shape	0.60967026	0.01057993	0.0127159	0.1028588	0
		gamma	rate	-2.36584464	0.012715902	0.018572	0.1236249	0.0573488
	EC	llogis	shape	0.70456675	0.0078157	-0.001252	0.0884064	0
		llogis	scale	3.019106	-0.001252254	0.0054719	-0.014165	0.0726036
	EC+mFOLFOX6	llogis	shape	0.52870595	0.0080863	-0.003511	0.0899239	0
		llogis	scale	3.32042152	-0.003510881	0.0073846	-0.039043	0.076552
